# Comparative Analyses of Reproductive Caste Types Reveal Vitellogenin Genes Involved in Queen Fertility in *Solenopsis invicta*

**DOI:** 10.3390/ijms242417130

**Published:** 2023-12-05

**Authors:** Fenghao Liu, Fengchao Xu, Yikun Zhang, Yurui Qian, Guofeng Zhang, Longqing Shi, Lu Peng

**Affiliations:** 1 State Key Laboratory of Ecological Pest Control for Fujian-Taiwan Crops, Institute of Applied Ecology, Fujian Agriculture and Forestry University, Fuzhou 350002, China; fenghaod@163.com (F.L.); 15267322001@163.com (F.X.); 15839424238@163.com (Y.Z.); xlanhuahuahua@163.com (Y.Q.); windlycountry@outlook.com (G.Z.); 2 Ministerial and Provincial Joint Innovation Centre for Safety Production of Cross-Strait Crops, Fujian Agriculture and Forestry University, Fuzhou 350002, China; 3 Fujian Provincial Key Laboratory of Insect Ecology, Fujian Agriculture and Forestry University, Fuzhou 350002, China; 4 Rice Research Institute, Fujian Academy of Agricultural Sciences, Fuzhou 350018, China; shilongqing@faas.cn

**Keywords:** *Solenopsis invicta*, transcriptome, *Vgs*, oogenesis, fertility

## Abstract

The red imported fire ant (*Solenopsis invicta* Buren) is a social pest species with a robust reproductive ability that causes extensive damage. Identification of the genes involved in queen fertility is critical in order to better understand the reproductive biology and screening for the potential molecular targets in *S. invicta*. Here, we used the mRNA deep sequencing (RNA-seq) approach to identify differentially expressed genes (DEGs) in the transcriptomes of three reproductive caste types of *S. invicta*, including queen (QA) and winged female (FA) and male (MA) ants. The genes that were specific to and highly expressed in the queens were then screened, and the *Vg2* and *Vg3* genes were chosen as targets to explore their functions in oogenesis and fertility. A minimum of 6.08 giga bases (Gb) of clean reads was obtained from all samples, with a mapping rate > 89.78%. There were 7524, 7133, and 977 DEGs identified in the MA vs. QA, MA vs. FA, and FA vs. QA comparisons, respectively. qRT–PCR was used to validate 10 randomly selected DEGs, including *vitellogenin 2* (*Vg2*) and *3* (*Vg3*), and their expression patterns were mostly consistent with the RNA-seq data. The *S. invicta* Vgs included conserved domains and motifs that are commonly found in most insect Vgs. *SiVg2* and *SiVg3* were highly expressed in queens and winged females and were most highly expressed in the thorax, followed by the fat body, head, and epidermis. Evaluation based on a loss-of-function-based knockdown analysis showed that the downregulation of either or both of these genes resulted in smaller ovaries, less oogenesis, and less egg production. The results of transcriptional sequencing provide a foundation for clarifying the regulators of queen fertility in *S. invicta*. The functions of *SiVg2* and *SiVg3* as regulators of oogenesis highlight their importance in queen fecundity and their potential as targets of reproductive disruption in *S. invicta* control.

## 1. Introduction

The red imported fire ant, *Solenopsis invicta* Buren (Hymenoptera: Formicidae), native to the Paraná River basin, South America, is globally recognised as one of the 100 most dangerous invasive species [1,2,3]. In recent years, with climate change and global economic development [4], its distribution has expanded to include tropical and subtropical areas of the Americas, India, Africa, Pacific islands, and so on, and it has invaded more than 10 provinces and cities in China [5]. These ants are extremely aggressive and territorial; for example, they attack other vertebrate or invertebrate animals to negatively affect the ecological balance [4,6], eat the seeds and roots of crops [7], and destroy power and communication facilities [8]. Moreover, when humans or livestock are attacked by *S. invicta*, they experience red and swollen skin accompanied by burning, and allergic individuals may develop symptoms such as pus-filled blisters, itching, anaphylactic shock, and even death [2,8,9,10].

As a typical social insect, *S. invicta* has colonies with a complete hierarchy and labour division, including reproductive ants, such as queens and winged females and males, and workers responsible for foraging, nesting, defence, and conservation [11]. Among them, reproductive ants are the key to maintaining the population. After the emergence of winged female and male ants, they will start mating flights and then land to establish new ant nests. After mating, the female ants remove their wings and become new queens [12]. The queen, also known as the functional queen, is the core of the ant colony, with a lifespan of 6–7 years. They control the entire ant colony by laying eggs and regulating the physiology and behaviour of worker and reproductive ants by releasing pheromones [13]. There are two types of *S. invicta* colonies, those with a single queen and those with multiple queens. The former type of queen can lay up to 800 eggs per day, while the latter can produce a total of 1500 to 5000 eggs per day in a colony [14]. The suppression of queen functions can lead to colony extinction. Therefore, the strong reproductive ability and special reproductive mode of *S. invicta* are important factors in its rapid colonisation and expansion. However, the molecular mechanism of *S. invicta* fertility has yet to be elucidated.

Vitellin (Vn) is the major egg yolk protein and is of great significance for the reproductive success of most oviparous species (e.g., insects), as it provides nutrition and energy for egg maturation and embryonic development [15,16]. Typically, Vn deposition is mediated by the yolk precursor protein vitellogenin (Vg) [17]. Vg is synthesised in the fat body, followed by a series of post-translational processes to form an oligomeric mature Vg, which is secreted into the haemolymph and taken up by oocytes via receptor-mediated endocytosis [18,19]. As such, Vg is essential for the fertility of oviparous animals. Additionally, *Vgs* have also been proven to regulate the labour division, social behaviour, and antioxidant capacity of social insects [20,21]. The initiation of Vg synthesis in insects is group-specific. For example, Vg synthesis in butterflies (Lepidoptera) begins in the adult stage [22]. In moths, however, it varies according to whether the adults feed or not. For example, a silkworm that does not feed in the adult stage starts from a prepupa during cocoon formation [23]. However, Vg synthesis in species with adult feeding is divided into two types: one is initiated in the cryptic adult stage [24], such as in *Manduca sexta*, and the other starts in the adult stage, such as in *Helicoverpa zea* [25]. For *S. invicta*, the activation of vitellogenin synthesis in different social types and tissues remains unclear.

The advent of high-throughput sequencing technologies has provided an opportunity to comprehensively examine all genes or a subset of functionally active genes in the genome of a specific species or tissue [26,27]. These techniques, especially transcriptomic analysis, are essential in order to better understand the differences in insect fecundity between males and females and the mechanisms underlying these differences. In this study, transcriptome sequencing was performed on queens and winged female and male ants of *S. invicta* to provide a genome-wide overview of specific gene expression in the different reproductive caste types. The genes that were specific to and highly expressed in the queens were then screened, and the *Vg2* and *Vg3* genes were chosen as targets to better understand their functions in oogenesis and fertility through an RNA interference (RNAi)-based loss-of-function approach. The results can lay a foundation for analysing the reproductive function of queens and designing “biological insecticides” that target the disruption of *S. invicta* reproduction.

## 2. Results

### 2.1. Transcriptomic Analyses

**Figure 1:**
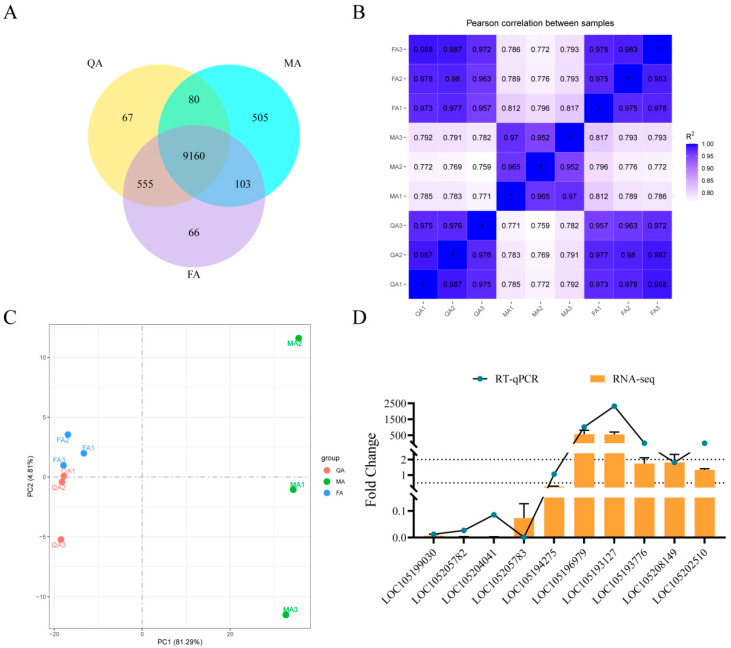
Principal component analysis and qPCR-based validation of transcriptomic data. (**A**) Venn diagram showing the expression of different genes in three samples. (**B**) Correlation analyses showing the relationship between biological replicate samples. (**C**) Scatter plots indicating PC1 and PC2 from the principal component analysis. (**D**) qPCR-based validation. QA: queens, FA: winged female ants, MA: winged male ants; the same below.

A minimum of 6.08 giga bases (Gb) of clean reads was obtained in all samples with Q20 percentages > 96.51%, while all of the GC contents ranged from 41.54 to 42.73% (Table S1). Clean reads from each sample were compared to the reference genome in the NCBI database, with a mapping rate > 89.78% and a unique mapping rate > 88.18% (Table S1). In total, 17,260 genes were identified in the samples from QA, FA, and MA. Of these, 9160 genes were coexpressed in all three samples, and among which 67, 66, and 505 genes were specifically expressed in the QA, FA, and MA, respectively (Figure 1A, Table S2). RNA-seq data among the three biological replicates were highly correlated (R^2^ > 0.95, Pearson correlation) (Figure 1B). Additionally, the samples for each social type (QA, FA, and MA) were well clustered along PC1 and PC2, further confirming that there was good repeatability in each biological replicate (Figure 1C). Subsequently, 10 genes were randomly selected from all samples for qPCR-based verification, and their expression patterns were consistent with the transcriptomic results (Figure 1D).

### 2.2. Differential Expression Analysis

**Figure 2:**
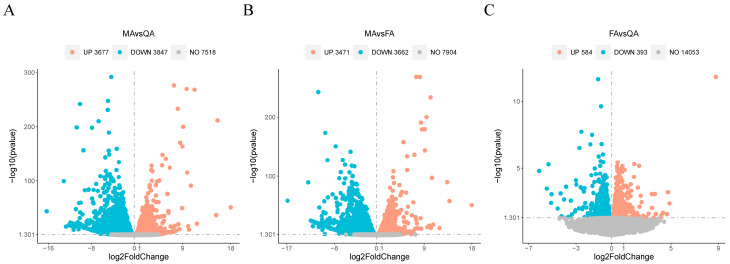
Analysis of differentially expressed genes (DEGs). (**A**) DEGs between MA and QA samples. (**B**) DEGs between MA and FA samples. (**C**) DEGs between FA and QA samples. Volcano map showing the DEGs between pairs of groups of samples (*p* value ≤ 0.05 and |log2 (fold change)|≥ 1).

Differentially expressed genes (DEGs) were identified using these quantitative transcriptomic data by comparing pairs of the groups of samples. There were 7524 DEGs when comparing MA and QA samples (3847 upregulated, 3677 downregulated), 7133 when comparing MA and FA samples (3662 upregulated, 3471 downregulated), and 977 when comparing FA and QA samples (393 upregulated, 584 downregulated) (Figure 2, Table S3). The number of DEGs between FA and QA was significantly lower than that between MA and QA or MA and FA; thus, it was considered that FA and QA might contain important potential regulators of female fertility, such as vitellogenin genes. Among them, *SiVg1* was expressed in all social types, *SiVg2* was specifically expressed in winged female ants and queens, and *SiVg3* was specifically expressed in queens (Table S3).

### 2.3. Metabolic Pathways Based on KEGG Analysis

**Figure 3:**
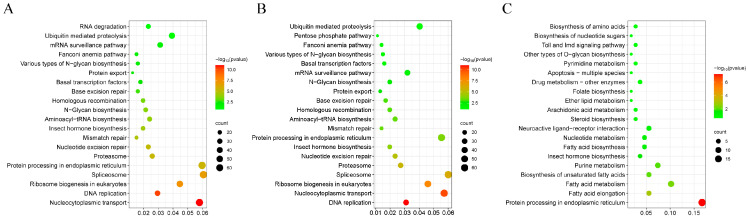
Functional classification of the differentially expressed genes (DEGs) based on KEGG analysis. The top 20 pathway-based gene numbers are displayed; (**A**) MA vs. QA, (**B**) MA vs. FA, (**C**) FA vs. QA. (**A**,**C**) enrichment based on the upregulated genes in QA; (**B**) enrichment based on the upregulated genes in FA. The horizontal axis is the gene ratio; the sizes and colours of the dots represent the gene number and *p* value, respectively; *p* value ≤ 0.05.

A total of 3847 and 393 upregulated genes in QA for MA vs. QA and FA vs. QA, respectively, as well as 3662 upregulated genes in FA for MA vs. FA, were matched to 118, 74, and 118 predicted KEGG pathways, respectively (Table S4). Based on gene numbers, the top 20 ‘highly enriched’ pathways are presented in Figure 3. Upregulated genes in QA and FA showed similar enrichment when the groups were compared with MA, and the primary KEGG pathways included nucleocytoplasmic transport (n = 65, 61), DNA replication (n = 33), insect hormone biosynthesis (n = 22, 21), and ribosome biogenesis in eukaryotes (n = 49) (Figure 3A,B). These predicted pathways will be of significance for future studies on the reproductive functions of *S. invicta*. Interestingly, upregulated genes in QA compared with FA were also significantly enriched in fatty acid elongation, metabolism, and biosynthesis (n = 6, 11, and 5, respectively), neuroactive ligand–receptor interaction (n = 6), folate biosynthesis (n = 2), and so on (Figure 3C).

### 2.4. Identification and Analysis of SiVgs

**Figure 4:**
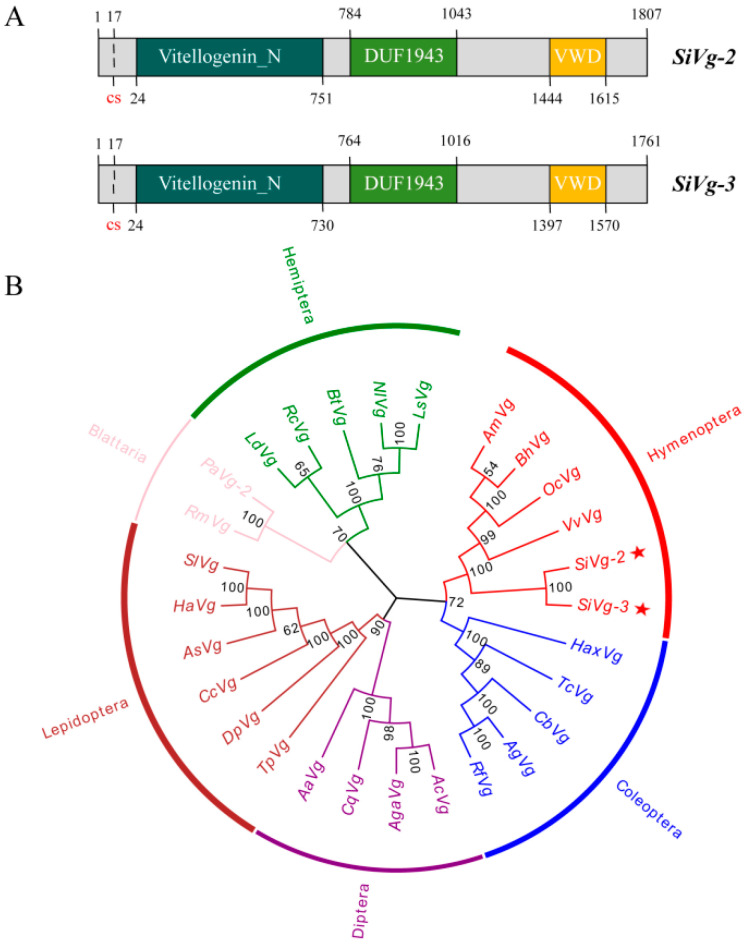
Sequence structure and phylogenetic tree of insect *VgRs*. (**A**) Diagrammatic comparison of typical domains between *SiVg2* and *SiVg3*. Cs indicates the signal peptides transported by the Sec translocon. (**B**) Phylogenetic tree of insect *Vgs* based on the neighbour-joining (NJ) method with a bootstrap value from 1000 replicates. The asterisk highlights the *S. invicta Vgs*. Sequences were deposited in the GenBank database, which included the *Vgs* of *Rhyparobia maderae* (*RmVg*, BAB19327.1), *Periplaneta americana* (*PaVg*, BAA86656.1), *Bemisia tabaci* (*BtVg*, ADU04393.1), *Lethocerus deyrollei* (*LdVg*, BAG12118.1), *Riptortus clavatus* (*RcVg*, AAB72001.1), *Nilaparvata lugens* (*NlVg*, BAF75351.1), *Laodelphax striatella* (*LsVg*, AGJ26478.1), *Tribolium castaneum* (*TcVg*, XP_971398.1), *Harmonia axyridis* (*HaVg*, ASO96848.1), *Colaphellus bowringi* (*CbVg*, AMK38869.1), *Anthonomus grandis* (*AgVg*, AAA27740.1), *Rhynchophorus ferrugineus* (*RfVg*, ALN38803.1), *Vespula vulgaris* (*VvVg*, AER70365.1), *Osmia cornifrons* (*OcVg*, AIU68826.1), *Apis mellifera* (*AmVg*, CAD56944.1), *Bombus ignitus* (*BiVg*, ACQ91623.1), *Bombus hypocrita* (*BhVg*, ACU00433.1), *Anopheles culicifacies* (*AcVg*, AEO51020.1), *Anopheles gambiae* (*AgVg*, AAF82131.1), *Culex quinquefasciatus* (*CqVg*, XP_001857970.1), *Aedes aegypti* (*AaVg*, AAA18221.1), *Thitarodes pui* (*TpVg*, AWJ95280.1), *Cadra cautella* (*CcVg*, ALN38805.1), *Danaus plexippus* (*DpVg*, OWR44310.1), *Helicoverpa armigera* (*HaVg*, AGL08685.1), *Spodoptera litura* (*SlVg*, ABU68426.1), *Lymantria dispar* (*LdVg*, AAC02818.1), *Bombyx mori* (*BmVg*, NP_001037309.1), *Actias selene* (*AsVg*, ADB94560.1), *Cnaphalocrocis medinalis* (*CmVg*, AEM75020.1), *Plutella xylostella* (*PxVg*, MN539627), and *Solenopsis invicta* (*SiVg*, NM_001304584.1, NM_001304585).

*SiVg2* and *SiVg3* had an open reading frame (ORF) with 5424 and 5286 base pairs (bp that encoded 1807 and 1761 amino acids (aa, respectively (Figure S1). The theoretical molecular weight was estimated at 23.76 and 20.10 kDa with isoelectric points of 7.0 and 8.4 for *SiVg2* and *SiVg3*, respectively. Three conserved domains were found in both *SiVgs*, including the Vg-N, VWD, and DUF1943 domains (Figure 4A). There were signal peptides transported by the Sec translocon in the N-terminus of *SiVg2* and *SiVg3* (Figure 4A). In addition, nine putative glycosylation sites (NXS/T) and many potential phosphorylation residues were found in the *SiVg2* and *SiVg3* sequences, respectively (Figure S2). Phylogenetic analysis showed that *Vgs* of different orders were highly conserved, with *SiVg2* and *SiVg3* belonging to the clade of Hymenoptera species, which formed a separate subclade (Figure 4B).

### 2.5. Expression Profile of SiVgs

**Figure 5:**
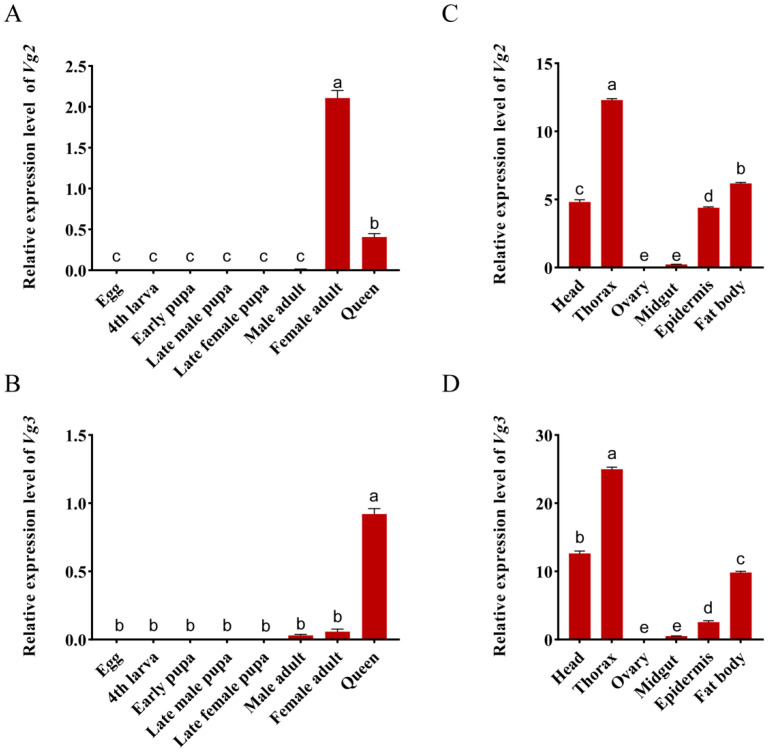
Developmental and tissue-specific expression profiles of *SiVgs.* The expression profiles of *SiVg* transcripts were analysed using qRT–PCR. (**A**,**B**) The expression profiles of *SiVg2* and *SiVg3* in the different developmental stages, respectively. (**C**,**D**) The expression profiles of *SiVg2* and *SiVg3* in the different tissues, respectively. The mRNA level was normalised relative to the *ef1-beta* level in qRT–PCR analysis. Data represent three biological replicates and each replication includes four technical replicates. The bars show the mean ± SE. Different letters indicate significant differences detected using one-way ANOVA with Tukey’s multiple range test for multiple comparisons at the *p* < 0.05 level.

The developmental expression profiles showed that *SiVg2* was specifically expressed in winged female ants and queens, where the expression level of the former was higher than that of the latter (*F*_7,16_ = 412.219, *p* < 0.05), and *SiVg3* had significantly higher expression in queens (*F*_7,16_ = 411.836, *p* < 0.05) (Figure 5A,B). Tissue-specific expression profiles showed that *SiVg2* and *SiVg3* were highly expressed in the thorax, followed by the fat body, head, and epidermis, and the expression levels were negligible in the midgut and ovary (*F*_2,12_ = 2704.913, *p* < 0.05) (*F*_5,12_ = 1955.393, *p* < 0.05) (Figure 5C,D).

### 2.6. The Dynamics of SiVg Expression and Oogenesis

**Figure 6:**
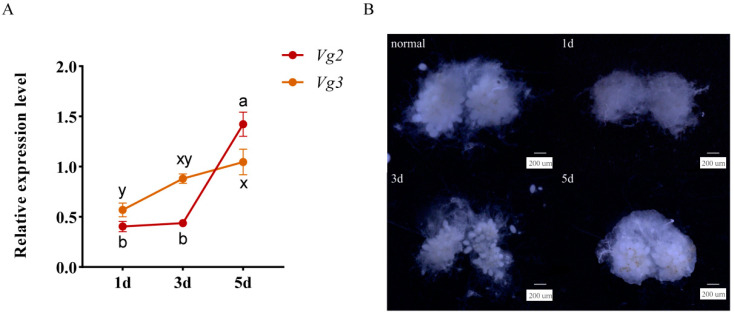
*SiVg* expression and oogenesis after isolation of the queens. (**A**) The expression profiles of *SiVg* transcripts were analysed using qRT–PCR. The mRNA level was normalised relative to the *ef1-beta* level in qRT–PCR analysis. Data represent three biological replicates and each replication includes four technical replicates. The bars show the mean ± SE. Different letters indicate significant differences detected using one-way ANOVA with Tukey’s multiple range test for multiple comparisons at the *p* < 0.05 level. (**B**) Ovary development was observed using a DMi8 stereomicroscope (Leica, Wetzlar, Germany); bar = 200 µm.

After the isolation of the queens, the expression levels of *SiVg2* and *SiVg3* gradually increased from Day 1 to Day 5 (Figure 6A) and were consistent with the oogenesis dynamics (Figure 6B). The number of eggs in the ovary also gradually increased and returned to that observed in the normal state of egg maturation in the queens on Day 5 (Figure 6B). These results provide an important basis for selecting the time point of RNAi.

### 2.7. RNAi Effect of SiVgs

**Figure 7:**
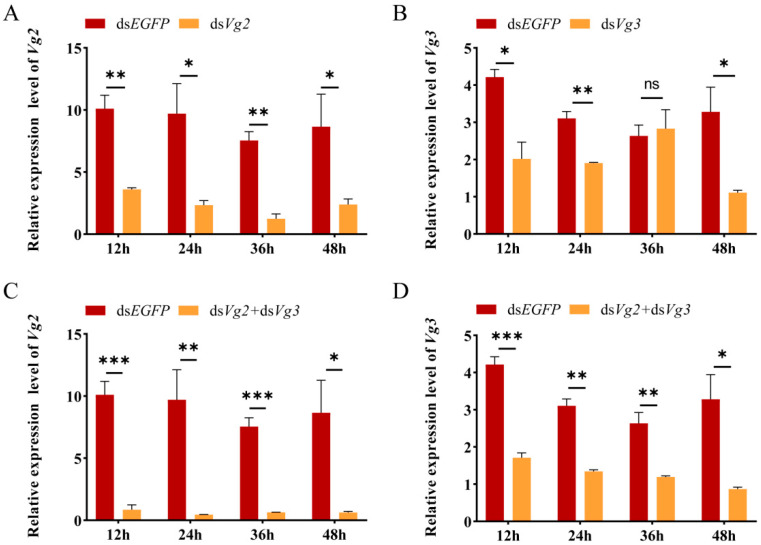
Silencing efficiency of *SiVg2* and *SiVg3* after gene-specific dsRNA injection. (**A**) Silencing efficiency of *SiVg2* after ds*Vg2* injection. (**B**) Silencing efficiency of *SiVg3* after ds*Vg3* injection. (**C**) Silencing efficiency of *SiVg2* after ds*Vg2* and ds*Vg3* injection. (**D**) Silencing efficiency of *SiVg3* after ds*Vg2* and ds*Vg3* injection. The expression levels of *SiVgs* transcripts were analysed using qRT-PCR. The mRNA level was normalised relative to the *ef1-beta* level in qRT-PCR analysis. Data represent three biological replicates and each replication includes four technical replicates. The data shown are the means ± SEs. Asterisks indicate significant differences detected using a two-tailed *t* test; ns *p* > 0.05, * *p* < 0.05, ** *p* < 0.01, and *** *p* < 0.001.

After ds*Vg2* injection for 12–48 h, the mRNA expression level of *SiVg2* in queens was significantly suppressed by 64.3–83.5% compared to that in the ds*EGFP* control group (12 h: *t* = 6.042, *df* = 4, *p* = 0.004; 24 h: *t* = 3.011, *df* = 4, *p* = 0.04, 36 h: *t* = 7.801, *df* = 4, *p* = 0.004; 48 h: *t* = 3.587, *df* = 4, *p* = 0.023) (Figure 7A). However, the expression level of *SiVg3* was significantly reduced by 66.2–38.7% after ds*Vg3* injection for 12, 24, and 48 h, while there was no significant difference at 36 h between the ds*Vg3* and ds*EGFP* groups (12 h: *t* = 4.435, *df* = 4, *p* = 0.011; 24 h: *t* = 6.467, *df* = 4, *p* = 0.003, 36 h: *t* = 0.334, *df* = 4, *p* = 0.755; 48 h: *t* = 3.790, *df* = 4, *p* = 0.019) (Figure 7B). After combined injection of ds*Vg2* and ds*Vg3* for 12–48 h, the expression levels of the *SiVg2* and *SiVg3* genes were significantly lower than those of the control group injected with ds*EGFP*. The suppression efficiency of *SiVg2* ranged from 91.5 to 95.2%, while that of *SiVg3* ranged from 54.7 to 73.6% (*SiVg2*, 12 h: *t* = 8.137, *df* = 4, *p* = 0.001; 24 h: *t* = 3.822, *df* = 4, *p* = 0.019, 36 h: *t* = 9.705, *df* = 4, *p* = 0.001; 48 h: *t* = 3.076, *df* = 4, *p* = 0.037; *SiVg3*, 12 h: *t* = 10.178, *df* = 4, *p* = 0.001; 24 h: *t* = 9.310, *df* = 4, *p* = 0.008, 36 h: *t* = 4.899, *df* = 4, *p* = 0.008; 48 h: *t* = 3.635, *df* = 4, *p* = 0.022) (Figure 7C,D).

### 2.8. The Effects of SiVg2 and SiVg3 Knockdown on Reproduction

**Figure 8:**
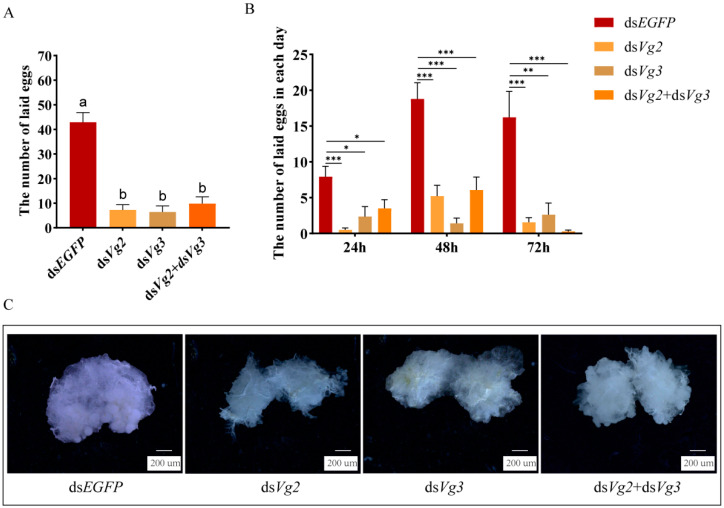
The effects of *SiVg2* and *SiVg3* knockdown on ovary development and reproduction. (**A**) Total egg production within 72h after dsRNA injection. The data shown are the mean ± SE. Different letters indicate significant differences detected using one-way ANOVA with Tukey’s multiple range test for multiple comparisons at the *p* < 0.05 level. (**B**) Daily egg production within 72h after dsRNA injection. Asterisks indicate significant differences detected using a two-tailed *t* test; * *p* < 0.05, ** *p* < 0.01, and *** *p* < 0.001. (**C**) Ovary development was observed using a DMi8 stereomicroscope (Leica, Wetzlar, Germany); bar = 200 µm.

The mean number of eggs laid per queen within 72 h significantly decreased from 43.3 under ds*EGFP* injection to 7.3 under ds*Vg2* injection, 6.4 under ds*Vg3* injection, and 9.9 under ds*Vg2* and ds*Vg3* injection; however, there was no significant difference between the latter three (*F*_4,51_ = 28.120, *p* < 0.05) (Figure 8A). Additionally, with the injection of ds*Vg2* or ds*Vg3* alone, as well as ds*Vg2* and ds*Vg3* together, the average number of eggs laid per day was significantly lower than that in the control group injected with ds*EGFP*, respectively (ds*Vg2*, 24 h: *t* = 4.996, *df* = 26, *p* = 0.00034; 48 h: *t* = 4.963, *df* = 26, *p* = 0.00037, 72 h: *t* = 3.967, *df* = 26, *p* = 0.0005; ds*Vg3*, 24 h: *t* = 2.741, *df* = 26, *p* = 0.0109; 48 h: *t* = 7.276, *df* = 26, *p* = 0.0000, 72 h: *t* = 3.414, *df* = 26, *p* = 0.0021; ds*Vg2 +* ds*Vg3*, 24 h: *t* = 2.321, *df* = 26, *p* = 0.028; 48 h: *t* = 4.383, *df* = 26, *p* = 0.0001, 72 h: *t* = 4.378, *df* = 26, *p* = 0.0001) (Figure 8B). The knockdown of ds*Vg2* and ds*Vg3* resulted in smaller ovaries and less oogenesis (Figure 8C).

## 3. Discussion

The advent of high-throughput sequencing technologies has facilitated the comprehensive detection of all genes or functionally active gene subsets in the genome of a specific species or tissue [26,27,28]. These techniques, especially comparative transcriptome analysis, have become important for understanding the molecular mechanisms underlying sexual differences in insect fecundity, such as in *Drosophila melanogaster* [29] and *Anopheles gambiae* [30,31,32]. Recently, the molecular mechanisms of reproductive regulation in social Hymenoptera insects, such as the honey bee and *Crematogaster osakensis*, have gradually become the focus of study. The red imported fire ant is a social pest species with a robust reproductive ability that causes extensive damage. However, relatively little attention has been given to *S. invicta*, whereby the reproduction of which is under complex regulation, involving endogenous and environmental factors. The identification of the genes involved in queen fertility is critical in order to better understand the reproductive biology and screening for the potential molecular targets in *S. invicta*.

Here, a high-quality transcriptomic sequencing analysis was conducted to clarify the gene expression differences between the three reproductive caste types of *S. invicta*, and RNAi technology was used to explore the molecular mechanism through which *Vg2* and *Vg3* mediate the reproductive development of queens. We found that the number of differentially expressed genes between MA and FA was much higher than that between FA and QA, suggesting that differences between males and females can be attributed to the evolution of differential gene expression in the two sexes [32,33] and indicating that FA and QA might include many important potential regulators of female fertility [30,34]. Three *SiVgs* were detected in the transcriptome, which is consistent with the results reported by Tufail and Takeda [18], but only *SiVg2* and *SiVg3* were specifically expressed in FA and QA, which are the reproductive females. Therefore, we suggest that *SiVg2* and *SiVg3* are closely related to female reproduction [16,35]. KEEG analysis showed that DEGs in QA were significantly enriched in the pathways of insect hormone biosynthesis, folate biosynthesis, and neuroactive ligand–receptor interaction, suggesting that these genes are closely related to the reproduction and population control of queens [36,37]. Additionally, upregulated DEGs in QA and FA compared with MA were significantly enriched in the nucleocytoplasmic transport pathway, which is of central importance in the regulation of germ cell differentiation, essential for fertility [38].

We further analysed the molecular characteristics and functions of *Vg2* and *Vg3* in *S. invicta* queens. Both *SiVg2* and *SiVg3* contained three typical conserved domains, Vg-N, DUF1943, and VWD, consistent with those of *Vgs* in most insect species [16,17,39]. As a lipoprotein domain, Vg-N is mainly responsible for the activity of lipid transport [16], the presence of DUF1943 might reduce lipid binding [40], and VWD helps *Vg* function as a pattern recognition receptor of microbes [41] and binding partner of sperm proteases [42]. Some putative glycosylation sites were found in *SiVg2* and *SiVg3*, which improved oocyte membrane recognition [43,44] as well as the transport and uptake efficiency of *Vgs* [18,45]. There were many putative glycosylation sites and potential phosphorylation residues found in *SiVg2* and *SiVg3*, which helped to promote the solubility of *Vg* [46]. The phosphate moieties have a negative charge and may work in receptor binding, as reported by Sappington et al. [47].

*SiVg2* and *SiVg3* were highly expressed only in FA and QA, and the pattern was almost synchronised with vitellogenesis and ovary development in *S. invicta*. Similar expression profiles have been found in other insects, such as *Nilaparvata lugens* [48], *P. xylostella* [16], and *Zeugodacus cucurbitae* [49]. Tissue expression showed that both *SiVg2* and *SiVg3* were mainly expressed in the thorax, fat body, and head, and presented negligible levels in the midgut and ovary. For most insects, the fat body is the main site of *Vg* synthesis, where *Vg* transcription levels are the highest [16,49,50,51]; however, we found that *SiVg2* and *SiVg3* were highly transcribed in the head and thorax, especially the thorax. There are two possible reasons for this: On the one hand, fat body cells may exist in the thorax and head [52,53], which also indicates that the high expression of *Vg* in fat body cells of the head and thorax promotes the longevity of queen bees. On the other hand, *Vgs* may play pleiotropic roles in insect species, especially in social insects such as bees [54,55], including in climate adaptation [56], behaviour construction [57], labour division [20], food transformation [58], and anti-stress and antioxidation [53,59].

Nutrients also play a key role in insect vitellogenesis [60]. We found that after queen isolation, the expression of *SiVg2* and *SiVg3* was downregulated and egg formation was hindered. This may be due to the specificity of the queens in maintaining their oviposition ability, which largely depends on the rumination of fourth-instar larvae to obtain amino acids and soluble proteins for laying eggs [13,61]. RNAi technology aims to clarify molecular functions by silencing specific genes [62]. Vitellogenin, as the major egg yolk precursor protein, is essential for ovarian and/or embryonic development in most invertebrate species [16,18]. Here, the inhibition of either *SiVg2* or *SiVg3* expression resulted in smaller ovaries and less oviposition, suggesting that *SiVg2* or *SiVg3* are both essential for queen fertility. Similarly, smaller ovaries and less egg production have also been reported in *Cimex lectularius* [39], *Aphis citricidus* [35], and *Z. cucurbitae* [48]. However, there are also some exceptions, and Zou et al. [16] showed that the knockout of *Vg* had no effect on ovary development, oocyte maturation, or oviposition; thus, it is speculated that *Vg* might not be the major egg yolk precursor protein in some lepidopteran species.

In summary, RNA-Seq technology was herein used to detect gene expression in three reproductive caste types. Among these, *SiVg2* and *SiVg3*, expressed specifically in QA and FA, were further studied to verify their importance as regulators of oogenesis in queens. These results suggest that *SiVg2* and *SiVg3* play a crucial role in queen fertility. Our work lays a foundation for understanding the molecular mechanisms of queen reproduction and screening, which are promising targets for the genetic control of *S. invicta*.

## 4. Materials and Methods

### 4.1. Insect Rearing

Nests of *S. invicta* were collected from the garden of Fujian Agriculture and Forestry University (26.08° N, 119.28° E) and were transported to an isolated laboratory using a plastic bucket with a lid (30 L). The inside of the plastic bucket was coated with talc powder to prevent ant escape. While the *S. invicta* built a stable nest in the bucket, water was injected into the bucket at 3 drops per second using a dropper, and we then gradually increased the drip rate. When the ant colonies floated from the soil and formed “ant rafts”, we used a long-handled slotted spoon to transfer them to a feeding box (300 mm × 200 mm × 100 mm) coated with talc powder on the inner wall [63]. The colony was fed with *Tenebrio molitor*, ham, and a 20% honey solution at 25 ± 2 °C, 65 ± 5% RH, and L:D = 16:12 h.

### 4.2. RNA Sequencing

Newly emerged winged female (FA) and male (MA) ants, as well as queens (QA), were collected for RNA extraction. Each sample was composed of 3 µg RNA from three individuals, with three biological replicates. An NEBNext^®^ Ultra^™^ RNA Library Prep Kit (Illumina, Greenland, NH, USA) was used for sequencing preparation according to the manufacturer’s instructions. RNA integrity (>6.5) was determined using an Agilent 2100 bioanalyzer (Agilent Technologies, Santa Clara, CA, USA), and then the Illumina NovaSeq 6000 (Illumina, Greenland, NH, USA) sequencing platform was used for paired-end sequencing. Finally, PCR products were purified using the AMPure XP system, and library quality was assessed using the Agilent Bioanalyzer 2100 system (Agilent, Santa Clara, CA, USA).

### 4.3. Quality Control and Read Mapping to the Reference Genome

Raw data (raw reads) in fastq format were first processed through fastp software. In this step, clean data (clean reads) were obtained by removing reads containing adapters, reads containing poly-N, and low-quality reads from the raw data. The Q20, Q30, and GC content of the clean data were calculated. All of the analyses were carried out using high-quality clean data. The index of the reference genome was built using HISAT2 v2.0.5, and paired-end clean reads were aligned to the reference genome using HISAT2 v2.0.5.

### 4.4. Differential Expression and KEGG Enrichment Analysis

Differential expression analysis of pairs of samples (three biological replicates per sample) was performed using the DESeq2 R package (1.20.0). The resulting *p* values were adjusted using Benjamini and Hochberg’s approach for controlling the false discovery rate. Genes with an adjusted *p* value ≤ 0.05 as detected using DESeq2 were considered to be differentially expressed. Additionally, the clusterProfiler R package (3.8.1) was used to test for statistical enrichment of differentially expressed genes in the Kyoto Encyclopedia of Genes and Genomes (KEGG) pathways (*p* value ≤ 0.05).

### 4.5. RNA Extraction and cDNA Synthesis

Total RNA was extracted from individuals or tissues using the Eastep^®^ Super Total RNA Extraction Kit (Promega, Madison, WI, USA), according to the manufacturer’s instructions. The RNA concentration and quality were measured using a Nano Vue spectrophotometer (GE-Health care, Chicago, IL, USA), and RNA purity was detected by 1% agarose gel electrophoresis. cDNA was synthesised using Fastking gDNA Dispelling RT SuperMix (Tiangen, Beijing, China) with an RNA concentration of 1000 ng/µL.

### 4.6. SiVg Cloning

The *SiVg* sequences obtained from the *S. invicta* Genome Database (https://www.ncbi.nlm.nih.gov/, accessed on 10 July 2023) were identified by common PCR using specific primers designed with SnapGene 4.3.6 (Table S5). The PCR was performed as follows: 94 °C pre-degeneration for 3 min, followed by 95 °C denaturation for 30 s, 58 °C annealing for 30 s, 72 °C for 26 s and 34 cycles, and, finally, 72 °C for 5 min for extension. All PCR products were purified and linked using the Hieff Clone^TM^ Zero TOPO-Blunt Simple Cloning Kit (Yeasen, Shanghai, China) for sequencing.

### 4.7. Molecular Characterisation and Phylogenetic Analysis

The fragments of *SiVgs* were assembled using DNAMAN 8.0 software. ORFfinder (https://www.ncbi.nlm.nih.gov/or#nder, accessed on 20 July 2023) was used to predict the open reading frames. Protein molecular weights and isoelectric points were calculated using ExPASy (The SIB Swiss Institute of Bioinformatics) (http://www.ExPASy.org, accessed on 20 July 2023). The SignalIP 6.0 Server was used to predict the signal peptide. Glycosylation sites and putative phosphorylation sites were predicted using the NetNGlyc 1.0 and NETPHOS 3.1 programs (http://www.cbs.dtu.dk/services, accessed on 20 July 2023). NCBI CD-Search (https://www.ncbi.nlm.nih.gov/Structure/cdd/wrpsb.cgi, accessed on 20 July 2023) was used to analyse the conserved domains. The phylogenetic tree was constructed with the neighbour-joining method using MEGA 7.0 and a bootstrap value of 1000 replicates.

### 4.8. qRT–PCR Analysis

For stage- and sex-specific expression profiles, all stages (eggs, 4th-instar larvae, early pupae, late pupae, adults, and queens) and both sexes (from late pupa to adult) were sampled (three samples of 100–150 mg (i.e., pooled individuals) per stage and sex). For tissue-specific expression patterns, 120 queens were dissected to extract RNA from the head, thorax, midgut, ovary, fat body, and epidermis tissues in DNase- and RNase-free water (Qiagen, FRA, Hilden, Germany) [28]. Each tissue of these individuals was then separately put in RNAprotect Tissue Reagent (RNAlaterTM, Qiagen, FRA, Hilden, Germany) and stored at −80 °C. RNA isolation and cDNA synthesis were conducted as described above. qRT–PCR was performed using the GoTaq^®^ qPCR Master Mix Kit (Promega, Madison, WI, USA) as follows: 95 °C, 30 s; 95 °C, 5 s; and 60 °C, 30 s, for 50 cycles. *ef1-beta* was used as the internal reference to normalise the gene expression levels according to the comparative Ct method (2^−ΔΔct^). The specific primers used for qPCR are listed in Table S5. Three biological replicates and four technical replicates were used for each sample.

### 4.9. Oogenesis Process of Queens

Several queens were collected and treated in isolation (no other instars or types of social individuals) and fed only 20% honey solution for nutrition. After 2 days of isolation, the queens were returned to the normal colony, and then individuals were collected on the 1st, 3rd, and 5th days. Some of them were used to observe oogenesis, with 10 queens in each subgroup; some of them were used to detect the transcription levels of *SiVg2* and *SiVg3*, with 3 queens per time point.

### 4.10. RNA Interference

The unique nucleotide regions of *SiVg2* and *SiVg3* were amplified using gene-specific primers (Table S5) containing T7 RNA polymerase promoters. The dsRNA of *SiVg2* and *SiVg3* was synthesised and purified using a T7 RiboMAX^TM^ Express RNAi System (Promega, Madison, WI, USA) according to the standard protocol. After purification, ds*SiVg2* and ds*SiVg3* were quantified and qualified using a Nano Vue spectrophotometer (GE-Health care) and agarose gel electrophoresis. Approximately 4 μg of ds*Vg2*, ds*Vg3*, ds*Vg2* + ds*Vg3*, and ds*EGFP* was separately injected into the abdomens of queens on the first day after isolation using a Nanoject III Auto-Nanoliter Injector (Drummond Scientific, Broomall, PA, USA) and ds*EGFP* was injected as a negative control. Each injected queen was kept with 20 workers and 2 pupae in a disposable Petri dish (60 mm) and nutrition was provided via ham sausage and 20% honey solution. Injected queens were collected after 12, 24, 36, and 48 h for gene silencing efficiency determination and *ef1-beta* was used as the internal reference gene. Each sample consisted of three biological replicates.

### 4.11. Phenotypic Observation and Bioassays

Ten ovaries were dissected from queens at 24, 48, and 72 h after dsRNA injection, as previously described in Section 4.9, and rinsed with PBS 3 times. Ovary and egg morphology was observed and photographed using a DMi8 stereomicroscope (Leica, Wetzlar, Germany). Additionally, the total numbers of eggs laid by each queen in three days and on each day were recorded. Each Petri dish was removed and replaced with a new dish every 24 h.

### 4.12. Statistical Analysis

Multiple comparisons were performed using one-way analysis of variance (ANOVA) and Tukey’s multiple range test (*p* < 0.05). The comparisons between *dsSiVgs* and *dsEGFP* strains were conducted using an independent sample *t* test (*, *p* < 0.05; **, *p* < 0.01). Data analyses were completed using SPSS Statistics Software 26. (SPSS Inc., Chicago, IL, USA).

## Data Availability

The data presented in this study are available from the corresponding author upon reasonable request.

## Supplementary Materials

The following supporting information can be downloaded at: https://www.mdpi.com/article/10.3390/ijms242417130/s1.
