# Supplementary material for: Comparative Analyses of Reproductive Caste Types Reveal Vitellogenin Genes Involved in Queen Fertility in Solenopsis invicta
Source: Int J Mol Sci. 2023 Dec 5;24(24):17130. doi: 10.3390/ijms242417130 (PMC10743176; doi:10.3390/ijms242417130)
Supplement: Supplementary file 1 [file ijms-24-17130-s001.zip › Fig. S1/Amino acid sequence.pdf]

Amino acid sequence of the *SiVg2* gene.

```
               10        20        30        40        50        60
1         ATGTGGTTCCCTGTCACCCTTCTCTTTCTTGCCGGCGTGGCCGTGGCCACAAATAATCAC
1          M  W  F  P  V  T  L  L  F  L  A  G  V  A  V  A  T  N  N  H

               70        80        90       100       110       120
61        GAGCATGCCTGGGAAACGGGGAACGAGTATCAATATTCTGTATTCGGTCGAACATTGGCA
21         E  H  A  W  E  T  G  N  E  Y  Q  Y  S  V  F  G  R  T  L  A

              130       140       150       160       170       180
121       GGTGTTGACAAGTTGAAACGACAATATACTGGAATTCAATATAATGGTATTCTTACCATT
41         G  V  D  K  L  K  R  Q  Y  T  G  I  Q  Y  N  G  I  L  T  I

              190       200       210       220       230       240
181       CAAGTAAAATCACCGGAGTTATTACAGGCAAAGTTTGATAATCAACATTATGCTCATATA
61         Q  V  K  S  P  E  L  L  Q  A  K  F  D  N  Q  H  Y  A  H  I

              250       260       270       280       290       300
241       CACCAAGAATTGTCGAACGGTCCGGACGATTTCGACGATCCTAAGAATGTCAATTATAAA
81         H  Q  E  L  S  N  G  P  D  D  F  D  D  P  K  N  V  N  Y  K

              310       320       330       340       350       360
301       CGAATGCCCATGTCAGAAAAGCCGTTTGAGATCAAATTGAAGCATGGCATAATCCGGGAT
101        R  M  P  M  S  E  K  P  F  E  I  K  L  K  H  G  I  I  R  D

              370       380       390       400       410       420
361       TTGTTATTCGACCGTGATGTACCTACTTGGGAAGTGAATATGATGAAGGCCATCGTAGGT
121        L  L  F  D  R  D  V  P  T  W  E  V  N  M  M  K  A  I  V  G

              430       440       450       460       470       480
421       CAGCTGCAAGTCGATACTCAGGGCGAAAACGCGATAAATAGCAAGAGCATTCAGGTTCCT
141        Q  L  Q  V  D  T  Q  G  E  N  A  I  N  S  K  S  I  Q  V  P

              490       500       510       520       530       540
481       AGCGACGAGTCCTTTGCCGCCACGTTTAAAGCCATGGAGGACTCCGTCAGTGGCAAATGC
161        S  D  E  S  F  A  A  T  F  K  A  M  E  D  S  V  S  G  K  C

              550       560       570       580       590       600
541       GAGGTTCTCTATGAGATTACGCCGTTGACCGTAAACGAAATACAAGCGAAGCAAGATAGA
181        E  V  L  Y  E  I  T  P  L  T  V  N  E  I  Q  A  K  Q  D  R

              610       620       630       640       650       660
601       ATACCAATGCCGTCTTTACACAGTGACGGTAATCATTATGAAGTTAAAAAGTTGAAGAAT
201        I  P  M  P  S  L  H  S  D  G  N  H  Y  E  V  K  K  L  K  N

              670       680       690       700       710       720
661       TATGAGAGGTGCCAGGAGCGACAGCTTTATCATTACGGCTTCGACATTAAATCGGCGGGA
221        Y  E  R  C  Q  E  R  Q  L  Y  H  Y  G  F  D  I  K  S  A  G

              730       740       750       760       770       780
721       AAGAAATGGGCCGGACAAGATAAAGTCATTTCGCAATTATCCGTGACTGAAATGGTTATC
241        K  K  W  A  G  Q  D  K  V  I  S  Q  L  S  V  T  E  M  V  I

              790       800       810       820       830       840
781       TCAGGCGACTTGAAACGTTTCACTATTCAATCGACTGAGATGAAGAATGAGATAGCTGTC
261        S  G  D  L  K  R  F  T  I  Q  S  T  E  M  K  N  E  I  A  V

              850       860       870       880       890       900
841       CAACCTGAAACATCTGATTCTCCCATTGGCAATGTCTACACCATAACAAGATTAACTTTG
281        Q  P  E  T  S  D  S  P  I  G  N  V  Y  T  I  T  R  L  T  L

              910       920       930       940       950       960
901       AAAAAGAAGAACTCGATTTCTAACTCTTGGTTTGGACCACACGAAATAAGCAACCTCGAA
301        K  K  K  N  S  I  S  N  S  W  F  G  P  H  E  I  S  N  L  E

              970       980       990      1000      1010      1020
961       TCAACCGGAAACCTCGTATACACATTCAATAACCCTTTCTCTGACTCTGACAACCGAAGA
321        S  T  G  N  L  V  Y  T  F  N  N  P  F  S  D  S  D  N  R  R

             1030      1040      1050      1060      1070      1080
```

1021 GTGCGTCACCATAGCGTTAGCCAAAATTCCGAGCAAGAAAATTCTTCAGAATCTAGCAAA
341 V R H H S V S Q N S E Q E N S S E S S K

1090 1100 1110 1120 1130 1140
1081 AGCTCTTCCCAAAGCTCCAGCTCCAGTTCCTCAGCCAGTAGCTCCAGCAGCTCCAGCAGC
361 S S S Q S S S S S S S A S S S S S S S S

1150 1160 1170 1180 1190 1200
1141 TCCAGCAGCTCTAGTAGCTCCAGTAGTTCTAGCAGCTCCAGCAGCTCAAGCAGCGAGGAA
381 S S S S S S S S S S S S S S S S S S E E

1210 1220 1230 1240 1250 1260
1201 GAAAATGAAAATGTCGTGCAAACTAAAGCGGCATTACAGAACATTTTCCTGGCTCCCAAC
401 E N E N V V Q T K A A L Q N I F L A P N

1270 1280 1290 1300 1310 1320
1261 ATCCCGCTGTTACCCTATTTCATCGGCTACAAAGGAAAGACGATCTTGAAGTCTGATAAG
421 I P L L P Y F I G Y K G K T I L K S D K

1330 1340 1350 1360 1370 1380
1321 CAAAACGTTATGCAATTTGCCAAGAACCTAATTTCTGAAATAGCTGAAGAAGTACAGATT
441 Q N V M Q F A K N L I S E I A E E V Q I

1390 1400 1410 1420 1430 1440
1381 ACTTCTGAGGGATATGAAGCTACAATGGAAAAATATACAATTCTGAAGAAACTCCTTCGC
461 T S E G Y E A T M E K Y T I L K K L L R

1450 1460 1470 1480 1490 1500
1441 ACCATGAATCGCAAGCAGTACGCTGAGTTAGAACAATATGTACTCCAATTTAATAAAGGA
481 T M N R K Q Y A E L E Q Y V L Q F N K G

1510 1520 1530 1540 1550 1560
1501 TCAGATTCCCGCGCAAATGCTTGGACCACCTTCCGCGATGCTGTCTTACATGCCGGAACT
501 S D S R A N A W T T F R D A V L H A G T

1570 1580 1590 1600 1610 1620
1561 GGACCTGCTTACGTCACTATCGAGAATTGGATAAAGAGTGGGCAAGTTAAAGGCGCAGAG
521 G P A Y V T I E N W I K S G Q V K G A E

1630 1640 1650 1660 1670 1680
1621 GCGGCACGACTCCTCTCTCAACTTCCCAAGAACGTATATTTACCGACGCCGAATTATGTT
541 A A R L L S Q L P K N V Y L P T P N Y V

1690 1700 1710 1720 1730 1740
1681 CAAGCATTCTTCGAACTGATCAAGAGTCCAATGGTAACTCAGCAAGAATACGTAAATGTG
561 Q A F F E L I K S P M V T Q Q E Y V N V

1750 1760 1770 1780 1790 1800
1741 TCCGCGCCTATAGCACTTGCCGAATTGCTCCGTAACAGTTATATCGGCCAAAATTACTAT
581 S A P I A L A E L L R N S Y I G Q N Y Y

1810 1820 1830 1840 1850 1860
1801 CCGATATACAGTTTCGGCTTTATAACTCTGAAGAAAAACGATGAGGTAGTTGGCAAATAT
601 P I Y S F G F I T L K K N D E V V G K Y

1870 1880 1890 1900 1910 1920
1861 ATCAACTACCTGGCTAATCAACTGCAACAAGGCTACCAAGAAAACAACAGCCGAAAAATT
621 I N Y L A N Q L Q Q G Y Q E N N S R K I

1930 1940 1950 1960 1970 1980
1921 CAGACGTACATCTTTGCACTTGGTGTCACTGCCCATCCGAAGATTATCTCAGTCTTTGAG
641 Q T Y I F A L G V T A H P K I I S V F E

1990 2000 2010 2020 2030 2040
1981 CCATACTTGGAACACAGCCTGCCGGCGTCAACGTACCAACGTACACTCATGGTAGCTGCC
661 P Y L E H S L P A S T Y Q R T L M V A A

2050 2060 2070 2080 2090 2100
2041 CTGTCTGATCTAGCCAAAGTTCAACCAAAATTAGTTGGACCGATTTTCTATAAGCTCTAC
681 L S D L A K V Q P K L V G P I F Y K L Y

2110 2120 2130 2140 2150 2160
2101 TTGAACGAAAACGAGGCTCACGAAGTTCGTGCTATGGCAGTACATGAATTTATCTTGACC
701 L N E N E A H E V R A M A V H E F I L T

2170 2180 2190 2200 2210 2220
2161 GACCCACCTATGATTACGTTGCAACGCATAGCGAAAAACACTAACTACGATACGAGCAAA
721 D P P M I T L Q R I A K N T N Y D T S K

2230 2240 2250 2260 2270 2280
2221 CAGGTGAACGCTGTCGTAAAGAGCACGCTAGAGAGTCTCGTTCATACGAAGCGATCAGAA
741 Q V N A V V K S T L E S L V H T K R S E

2290 2300 2310 2320 2330 2340
2281 TGGCGACATCTTGCTAACAAGGCACGCAATGTTAGGTATCTAGTGACTTCAAATAATTAT
761 W R H L A N K A R N V R Y L V T S N N Y

2350 2360 2370 2380 2390 2400
2341 GGCAACTGGCATTCGAGTGGCTACCATCTAGACTTCCAAGATTGGCTTGTTAACGGACTT
781 G N W H S S G Y H L D F Q D W L V N G L

2410 2420 2430 2440 2450 2460
2401 TCTCTGCAAACCATTGCTGGTGACGATCTAATACCGAAATATGTATATGTCGGTGTAAAT
801 S L Q T I A G D D L I P K Y V Y V G V N

2470 2480 2490 2500 2510 2520
2461 AGCGTTTTCGATTTCCTTGATCAGCCCTCCGTGGAAGCAGGATACGGGCTTTCGAGTCAC
821 S V F D F L D Q P S V E A G Y G L S S H

2530 2540 2550 2560 2570 2580
2521 AGGCAGTTTTTCAATGAAATCAGTAAGCAGTGGTACTCTCATCAAGCCGACGATGAGCGA
841 R Q F F N E I S K Q W Y S H Q A D D E R

2590 2600 2610 2620 2630 2640
2581 CGAAGGTCACGCGTTGAGAAGCTAGCGCAAGCACTTCAAATCAAGGCCAAAGAGCAGAAT
861 R R S R V E K L A Q A L Q I K A K E Q N

2650 2660 2670 2680 2690 2700
2641 AATTTGGAAGGACATTTCTTGTTTAACTCGGTATACGATTCGGCGTTCTATCCTTACGAC
881 N L E G H F L F N S V Y D S A F Y P Y D

2710 2720 2730 2740 2750 2760
2701 AGACACAGAATTAGAGAGGCTGTTGCTGCGCTGAAACAATTCCTGAATGGAAACAACAAA
901 R H R I R E A V A A L K Q F L N G N N K

2770 2780 2790 2800 2810 2820
2761 CTGGAAGGATCTGCATTCAACAATTATGAAAACATAATGAGCTTTGCGAATGAAGACGGT
921 L E G S A F N N Y E N I M S F A N E D G

2830 2840 2850 2860 2870 2880
2821 CTGCCGTTTGTCTATACTTTGGATGCGCCAACATTTACAAAAGCTAAAGTTAATTTCAAA
941 L P F V Y T L D A P T F T K A K V N F K

2890 2900 2910 2920 2930 2940
2881 CAAGGAGGACGCACCGCGAACAGTGGCACTTTCCAAGCGTTAATCGCCAATAATGTACAG
961 Q G G R T A N S G T F Q A L I A N N V Q

2950 2960 2970 2980 2990 3000
2941 CAACAATTTGGTTTTGTAGCACTTTTCGAACATCAGAAATACATTGCTGGTATCAACAAC
981 Q Q F G F V A L F E H Q K Y I A G I N N

3010 3020 3030 3040 3050 3060
3001 AATCGAGTGCTGCGTGTACCTGTGGAGTATGACGTCGAATTCAATTCGAACAACGCGAAA
1001 N R V L R V P V E Y D V E F N S N N A K

3070 3080 3090 3100 3110 3120
3061 AACTTTGCGTTGAAGATTCGTCCGCAGAACTTACCTCGAATGGGTAATGAACTCAAACTG
1021 N F A L K I R P Q N L P R M G N E L K L

3130 3140 3150 3160 3170 3180
3121 TTGCACTACAGCGTCGTTCCCTTCACCACGCAACAAGACCTCCTTGATCTTAAACCTACA
1041 L H Y S V V P F T T Q Q D L L D L K P T

```
        3190      3200      3210      3220      3230      3240
3181    TCAAATGACAAAAATACGCGTCCTGTATTCACTTCACCAGTATACAAAACCACGGTACAA
1061     S  N  D  K  N  T  R  P  V  F  T  S  P  V  Y  K  T  T  V  Q

        3250      3260      3270      3280      3290      3300
3241    AAAGATACATTTTCCATCAAGGTAGAATCTGATAGTATTGGAAAAGAATCTGACACGGAA
1081     K  D  T  F  S  I  K  V  E  S  D  S  I  G  K  E  S  D  T  E

        3310      3320      3330      3340      3350      3360
3301    AGTTTTGTTGCGGACGTCTTGAGATTAACAAATGCTAATGACGATCATTATACCAAGATA
1101     S  F  V  A  D  V  L  R  L  T  N  A  N  D  D  H  Y  T  K  I

        3370      3380      3390      3400      3410      3420
3361    AGCACAATATTAACTTCTGATCAGATACAAAAATCTGAGGGACACATCACCATGACGTAT
1121     S  T  I  L  T  S  D  Q  I  Q  K  S  E  G  H  I  T  M  T  Y

        3430      3440      3450      3460      3470      3480
3421    GATACGGTGACAATTGGCGGTAATAATGATAACTCCGGACAATCGAGCGAAGAGATGGAA
1141     D  T  V  T  I  G  G  N  N  D  N  S  G  Q  S  S  E  E  M  E

        3490      3500      3510      3520      3530      3540
3481    AGCCTTCATTCCATAAGCTGGAAATCAAACAGTAAGGAGAGAAGGAAACAGATTGCAAAT
1161     S  L  H  S  I  S  W  K  S  N  S  K  E  R  R  K  Q  I  A  N

        3550      3560      3570      3580      3590      3600
3541    AACCTTAGCAAAGGTATCAAGTCAGGCGAAGTCTATATATTTGACGTAAGTTACAGCGTC
1181     N  L  S  K  G  I  K  S  G  E  V  Y  I  F  D  V  S  Y  S  V

        3610      3620      3630      3640      3650      3660
3601    CCAATGTTACATGAGAATGAGTATGTTTTTACCTTCGGTGGAATGAAGAGCAATTCAAAT
1201     P  M  L  H  E  N  E  Y  V  F  T  F  G  G  M  K  S  N  S  N

        3670      3680      3690      3700      3710      3720
3661    CAGAAGTTAAGAGGTTACTTTTATTGGAATTCTCATGCGCCGCAAGAGGTGAACTATGAA
1221     Q  K  L  R  G  Y  F  Y  W  N  S  H  A  P  Q  E  V  N  Y  E

        3730      3740      3750      3760      3770      3780
3721    GTTTGTTTCTCACATGAAATGCAATATGCACCACGTGCTACTCCTCTCAATTTCAAATAC
1241     V  C  F  S  H  E  M  Q  Y  A  P  R  A  T  P  L  N  F  K  Y

        3790      3800      3810      3820      3830      3840
3781    GCCCTTAAGAACTCACCAAGAGATGAATACAAGGCTGTATTGAAATATGGAAAGACCTGC
1261     A  L  K  N  S  P  R  D  E  Y  K  A  V  L  K  Y  G  K  T  C

        3850      3860      3870      3880      3890      3900
3841    GCTACAGGAAATAAAGTTGTTATCACCGGAAGCTCGTCGCAAAGTCAACAACTGAGGGAT
1281     A  T  G  N  K  V  V  I  T  G  S  S  S  Q  S  Q  Q  L  R  D

        3910      3920      3930      3940      3950      3960
3901    ATAATAGAGAACTCCAGCTTACCAAACAATGTCTGGAAGAGATTTCAAACGGGAAACAAA
1301     I  I  E  N  S  S  L  P  N  N  V  W  K  R  F  Q  T  G  N  K

        3970      3980      3990      4000      4010      4020
3961    GCAGTGGAAAATTGCATGAAGGCCAATGACATCGCCCAAATGAGAGATCAGATAGATGTT
1321     A  V  E  N  C  M  K  A  N  D  I  A  Q  M  R  D  Q  I  D  V

        4030      4040      4050      4060      4070      4080
4021    CAATTTGACCTATCCAACATTGTTCCTGAATCCGTCCGAAGATATGCTAAGAAGATTATT
1341     Q  F  D  L  S  N  I  V  P  E  S  V  R  R  Y  A  K  K  I  I

        4090      4100      4110      4120      4130      4140
4081    GAATATTTAGAAAAATACGTCTACAAAGTTTGTGACAATGTATCACGTGAGGAAGAATCC
1361     E  Y  L  E  K  Y  V  Y  K  V  C  D  N  V  S  R  E  E  E  S

        4150      4160      4170      4180      4190      4200
4141    GAAGAAAACACAATCAAAAATACACTGCTGTTTGCATCACCAGTCAACAGAATGTGGCCC
1381     E  E  N  T  I  K  N  T  L  L  F  A  S  P  V  N  R  M  W  P

        4210      4220      4230      4240      4250      4260
4201    AACTGGCTTCCGCAATCAGTGAGCGATATTACTTCTGGTTGGTCTCCCTCATTTAATTCC
```

```
1401     N  W  L  P  Q  S  V  S  D  I  T  S  G  W  S  P  S  F  N  S

             4270      4280      4290      4300      4310      4320
4261     TTTGGACCAAGTTCGGAATCACAGTGGCAAACAATGAGACTAAATGTAGCTGACTATCCG
1421     F  G  P  S  S  E  S  Q  W  Q  T  M  R  L  N  V  A  D  Y  P

             4330      4340      4350      4360      4370      4380
4321     GATGAATTTGAATCAGAAAAACAATCATGCACTCTCGATAAAGACAAGGTTTATACTTTT
1441     D  E  F  E  S  E  K  Q  S  C  T  L  D  K  D  K  V  Y  T  F

             4390      4400      4410      4420      4430      4440
4381     GATAACCAGCTCTATAATGTGCACCTAGGAAAATGTAAACACGTGCTTTTGACCACTTAT
1461     D  N  Q  L  Y  N  V  H  L  G  K  C  K  H  V  L  L  T  T  Y

             4450      4460      4470      4480      4490      4500
4441     CCGCAAGATTTCCATAATCGCAGAAATTATATTCCGGAAAACTCGAAAGTGGCTATCTTA
1481     P  Q  D  F  H  N  R  R  N  Y  I  P  E  N  S  K  V  A  I  L

             4510      4520      4530      4540      4550      4560
4501     GCTGAAGATGCGGACAATGACAGCAGGAATGTTTATATATGGTTGGGCAAACAAGAAATT
1501     A  E  D  A  D  N  D  S  R  N  V  Y  I  W  L  G  K  Q  E  I

             4570      4580      4590      4600      4610      4620
4561     AAACTAAAGAAAGCGGGTAATAATGTACAAGCTGTAGTAAACGGACAGAATGTCGAGATA
1521     K  L  K  K  A  G  N  N  V  Q  A  V  V  N  G  Q  N  V  E  I

             4630      4640      4650      4660      4670      4680
4621     TCGGACAAAGGCTATCAAAAGATTAATGGAAATGAAATCACTTTCGAAATCCTGAGCCTA
1541     S  D  K  G  Y  Q  K  I  N  G  N  E  I  T  F  E  I  L  S  L

             4690      4700      4710      4720      4730      4740
4681     CCGGACGACTCCCTGTCTGTCGTTTCGGAGAAGTATGGAATTAATGCCGTGTACGATGGA
1561     P  D  D  S  L  S  V  V  S  E  K  Y  G  I  N  A  V  Y  D  G

             4750      4760      4770      4780      4790      4800
4741     AAACGCGTTGTAATAAGTGCAAGTGATGCATATCGCAATGCCGTACGTGGTCTCTGCGGT
1581     K  R  V  V  I  S  A  S  D  A  Y  R  N  A  V  R  G  L  C  G

             4810      4820      4830      4840      4850      4860
4801     AACTTCGACTCCAGGCCCAACACCGATTTCGTCACTCCTAAGAACTGTCTCTTGACGAAA
1601     N  F  D  S  R  P  N  T  D  F  V  T  P  K  N  C  L  L  T  K

             4870      4880      4890      4900      4910      4920
4861     CCGGAAGAATTTGCCGCTACCTACGCTATGACTCAAGAAAATTGCCAAGGACCTGCTCCG
1621     P  E  E  F  A  A  T  Y  A  M  T  Q  E  N  C  Q  G  P  A  P

             4930      4940      4950      4960      4970      4980
4921     GAAAACAAACGAAGAGCCGAACAATCCACGTGCCATGAATTTCCAGAGAATGAACAGATG
1641     E  N  K  R  R  A  E  Q  S  T  C  H  E  F  P  E  N  E  Q  M

             4990      5000      5010      5020      5030      5040
4981     AACGTTATTAGCGATAGAGAAGCGGGTAGAATGATGACAGAGGGCGTAAATTGGGGCTAC
1661     N  V  I  S  D  R  E  A  G  R  M  M  T  E  G  V  N  W  G  Y

             5050      5060      5070      5080      5090      5100
5041     CACCAGGCTAACCGCAATAAAGAACATGGACGAGGTAATAAAAGCCATCAAAATAATAAG
1681     H  Q  A  N  R  N  K  E  H  G  R  G  N  K  S  H  Q  N  N  K

             5110      5120      5130      5140      5150      5160
5101     AAACAGTATCAGGCAAACTCGCAAGAGAGTGGATCAAGCGAGAGCCGAAACGATAAGAAG
1701     K  Q  Y  Q  A  N  S  Q  E  S  G  S  S  E  S  R  N  D  K  K

             5170      5180      5190      5200      5210      5220
5161     AAACACAACATTGTCTATCGCACAAGAGTTGTAGAAGAGGGTGACGAAATCTGTTTCACT
1721     K  H  N  I  V  Y  R  T  R  V  V  E  E  G  D  E  I  C  F  T

             5230      5240      5250      5260      5270      5280
5221     ACTACACCTCTGCCAGCATGCCGCCAAGGTGCCAGGCCCACGGAAAGATATCCGAAGAAG
1741     T  T  P  L  P  A  C  R  Q  G  A  R  P  T  E  R  Y  P  K  K

             5290      5300      5310      5320      5330      5340
```

```
5281     GCTGATCTTTATTGCATGCCGAGGAACGATCAATCTTTGGATCTCAAACGTAGGGTCGAG
1761      A  D  L  Y  C  M  P  R  N  D  Q  S  L  D  L  K  R  R  V  E

             5350      5360      5370      5380      5390      5400
5341     GATGGCGCTAATCCAGATTTTACTCGGAAGTCTGTCAGCAGAATGCAAGTCTTCCAAGTT
1781      D  G  A  N  P  D  F  T  R  K  S  V  S  R  M  Q  V  F  Q  V

             5410      5420
5401     CCTGTCTCTTGCAGCGCCGCATGA
1801      P  V  S  C  S  A  A  *
```

Amino acid sequence of the *SiVg3* gene.

```
                  10        20        30        40        50        60
1         ATGTGGTTCCCTGTCGTCCTTCTCTTACTTGTCGGCGTGGCCGTGGCCGTGCCTGACCAC
1          M  W  F  P  V  V  L  L  L  L  V  G  V  A  V  A  V  P  D  H

                  70        80        90       100       110       120
61        GAGCATGCCTGGGAACCGCAGAACGAGTATCAATACTCTGTATTCGTTCGGACACTGACT
21         E  H  A  W  E  P  Q  N  E  Y  Q  Y  S  V  F  V  R  T  L  T

                 130       140       150       160       170       180
121       GGTGTGGACACGTTGAAACAGCAATATACTGGAATTCAATTAAAGGGTGTTCTCGTCATT
41         G  V  D  T  L  K  Q  Q  Y  T  G  I  Q  L  K  G  V  L  V  I

                 190       200       210       220       230       240
181       CAAGTAAAATCAGAGGAGTTGTTGCAAGCGAAGTATATCAATCCACGATATGCTCATATA
61         Q  V  K  S  E  E  L  L  Q  A  K  Y  I  N  P  R  Y  A  H  I

                 250       260       270       280       290       300
241       CACCAAGAATTATCGAACGGTCCGTATTCCAAGATTCCTGAAGAGAATCTCGAATATCGC
81         H  Q  E  L  S  N  G  P  Y  S  K  I  P  E  E  N  L  E  Y  R

                 310       320       330       340       350       360
301       GATATACCCATGTCGGGAAAGCCATTTGAGATCAAGTTGAAGCACGGAGTGATCCGGGAT
101        D  I  P  M  S  G  K  P  F  E  I  K  L  K  H  G  V  I  R  D

                 370       380       390       400       410       420
361       TTATTATTCGATCGTAACGTACCTACTTGGGAGGTGAATATGCTCAAGGGTATCGTAGGT
121        L  L  F  D  R  N  V  P  T  W  E  V  N  M  L  K  G  I  V  G

                 430       440       450       460       470       480
421       CAGCTGCAGATCGACACTCAGGGCGAAAACGCGATAGATAGCCAGAGTACTCAGATTCCC
141        Q  L  Q  I  D  T  Q  G  E  N  A  I  D  S  Q  S  T  Q  I  P

                 490       500       510       520       530       540
481       TCCAACTCAGAGCCTTCTTCCGCTACGTTTAAAGCCATGGAGGATTCCGTCGGTGGCAAA
161        S  N  S  E  P  S  S  A  T  F  K  A  M  E  D  S  V  G  G  K

                 550       560       570       580       590       600
541       TGCGAGGTTCTATATGAGATTACGCCGTTGCCCCAACATGTAGCCCAAACGAGGCCAGAT
181        C  E  V  L  Y  E  I  T  P  L  P  Q  H  V  A  Q  T  R  P  D

                 610       620       630       640       650       660
601       AGAGTACCTATGTCGTCTGTACCCAGCAAAGGTCATCATTATGAAGTTAAGAAGTTGAAG
201        R  V  P  M  S  S  V  P  S  K  G  H  H  Y  E  V  K  K  L  K

                 670       680       690       700       710       720
661       AATTATGAGAAGTGCCAGGAGCGACAGCTCTACCATTACGGTATGGACGTTAAAATGACG
221        N  Y  E  K  C  Q  E  R  Q  L  Y  H  Y  G  M  D  V  K  M  T

                 730       740       750       760       770       780
721       AAGGAAAATATGATGAAACGAAATAAAGTTGTTTCGGAATTATCCACGACTCACATAGTT
241        K  E  N  M  M  K  R  N  K  V  V  S  E  L  S  T  T  H  I  V

                 790       800       810       820       830       840
781       ATCACGGGTACCTTGAAAAGTTTCACCATTCAATCTACTGAGATGAAGAACGAGATAACC
261        I  T  G  T  L  K  S  F  T  I  Q  S  T  E  M  K  N  E  I  T

                 850       860       870       880       890       900
841       GTTCAACCTGAATCATCCGATTCTCCCATTGGTACTGTCTACAGTATAACGAAATTAACT
281        V  Q  P  E  S  S  D  S  P  I  G  T  V  Y  S  I  T  K  L  T

                 910       920       930       940       950       960
901       TTGGCCAAAATAAACAAAATCTCCAACTCTTGGTTTGGACCACTCGAATTAAGCAACGTC
301        L  A  K  I  N  K  I  S  N  S  W  F  G  P  L  E  L  S  N  V

                 970       980       990      1000      1010      1020
961       GAGTCAACTGGAAATCTGGTGTACATATTTAATAATCCTTTCTCTGATTCCGAGCAGCGA
321        E  S  T  G  N  L  V  Y  I  F  N  N  P  F  S  D  S  E  Q  R
```

1030 1040 1050 1060 1070 1080
1021 AAAGTAGGTCAGCCGAGCATTAGTCGAAATTCCGAGCAAGAAAACTCTTTAGAGACCAAA
341 K V G Q P S I S R N S E Q E N S L E T K

1090 1100 1110 1120 1130 1140
1081 AAAAGATCTTTCCACAGTCACAGTTCCTCATCCAGTAGCTCTAGTAGTTCCAGCAGCGAG
361 K R S F H S H S S S S S S S S S S S S E

1150 1160 1170 1180 1190 1200
1141 GAAGAAAATGAATCTGTCATGCAATCTAAGGCGTCATTACGAAACATTTTCATGGCTCCG
381 E E N E S V M Q S K A S L R N I F M A P

1210 1220 1230 1240 1250 1260
1201 AACGTCCCGCTGTTACCTTACTTTATCGGTTTCAAAGGCAAGACGATCATGAAATCTGAT
401 N V P L L P Y F I G F K G K T I M K S D

1270 1280 1290 1300 1310 1320
1261 GAGCACAACGTTATGCAACTTGCCAAGGACTTACTTCTCCAAATAGCTAAAGAAATACAG
421 E H N V M Q L A K D L L L Q I A K E I Q

1330 1340 1350 1360 1370 1380
1321 AATCCTTCTGAAGGATATGAGAATACGCTGGAGAAATATGTAAACTTAAAGAATCTCATT
441 N P S E G Y E N T L E K Y V N L K N L I

1390 1400 1410 1420 1430 1440
1381 CGCACCATGGATCGCAAGCAGTACACTGAGTTAGAGCAATATGTATCCCAATTTAATAAG
461 R T M D R K Q Y T E L E Q Y V S Q F N K

1450 1460 1470 1480 1490 1500
1441 GCAACAGTGGAAGGCGAAAATGCTTGGTACACCTTACGCGATGCTGTCGTACATGCTGGA
481 A T V E G E N A W Y T L R D A V V H A G

1510 1520 1530 1540 1550 1560
1501 ACTGGACCTGCTTTTGTCACTATCGAAAATTGGCTAAAGAGTGGACAAGTTAAAGGCGAA
501 T G P A F V T I E N W L K S G Q V K G E

1570 1580 1590 1600 1610 1620
1561 GAGGCGGCAGAACTTCTTTCTAAAATTCCCAAAAGCGTTCACCAACCAACACCGGATTAT
521 E A A E L L S K I P K S V H Q P T P D Y

1630 1640 1650 1660 1670 1680
1621 ATCAAAGAATTCTTTAAACTGATTAAGAGTTCAGTGGTAACTCAACAAGAATACGTAAAT
541 I K E F F K L I K S S V V T Q Q E Y V N

1690 1700 1710 1720 1730 1740
1681 GTGTCCGCACCTCTAGCATTTGCTGAACTGTTACGCAATAATTATGTCGTCCCATCTTAC
561 V S A P L A F A E L L R N N Y V V P S Y

1750 1760 1770 1780 1790 1800
1741 TATCCGGTACACAGTTTCGGCCGTATGACTCTTAAGGGAAATGAAGAGATAGACAACTAT
581 Y P V H S F G R M T L K G N E E I D N Y

1810 1820 1830 1840 1850 1860
1801 ATTTCTTATTTGGCTAACCAATTGCAACAAGGCTACCTCGAAAACAATACTCAGAAAATT
601 I S Y L A N Q L Q Q G Y L E N N T Q K I

1870 1880 1890 1900 1910 1920
1861 CAGACATTCATCTTTGCACTTGGTGTCACTGCCCATCCGAAGATTATCTCAGTCTTTGAG
621 Q T F I F A L G V T A H P K I I S V F E

1930 1940 1950 1960 1970 1980
1921 CCATACTTAGAAGGCAAGCTGCCGACGACGAAATATCAACGTATGCTCATGGTGGCCGCT
641 P Y L E G K L P T T K Y Q R M L M V A A

1990 2000 2010 2020 2030 2040
1981 CTGTATGATCTATCCAGAGACATACCAAAATTAGTTGGACCAATTTTCTATAAGCTCTAC
661 L Y D L S R D I P K L V G P I F Y K L Y

2050 2060 2070 2080 2090 2100
2041 ATGAATGAAAACGAAGCTCACGAAGTTCGTTGCATGGCAGTACAGCAATTTATCCTGACA
681 M N E N E A H E V R C M A V Q Q F I L T

2110 2120 2130 2140 2150 2160
2101 GACCCGCCAATGATTACGTTGCAACGCGTAGCGAAATACACTAATTACGATCAGAGTGAT
701 D P P M I T L Q R V A K Y T N Y D Q S D

2170 2180 2190 2200 2210 2220
2161 CAGGTGAACTCTGCCGTGAAGAGTACGCTAAACAGCATCATTAATACGAAGCGACCGGAA
721 Q V N S A V K S T L N S I I N T K R P E

2230 2240 2250 2260 2270 2280
2221 TGGCGAAATCTCGCTAACAAGGCGCGCAGTGTCAGGTATCTAGTGAATCCAAAGAACTAT
741 W R N L A N K A R S V R Y L V N P K N Y

2290 2300 2310 2320 2330 2340
2281 GACACCTGGTACTCGAAGGGCTACTATATAGATTTTGAAAACTGGGTTTTCAAAGGACTT
761 D T W Y S K G Y Y I D F E N W V F K G L

2350 2360 2370 2380 2390 2400
2341 AATGTGAAAATGGTTGCTAGTAATGATGCCGTACTACCCAGATATGTATACGTTGGCCTT
781 N V K M V A S N D A V L P R Y V Y V G L

2410 2420 2430 2440 2450 2460
2401 GATAGTATTTTCAACTTCCTCCGTAAGCCCACTTTCGAAGTAGGATATGCGGTATCGAGC
801 D S I F N F L R K P T F E V G Y A V S S

2470 2480 2490 2500 2510 2520
2461 TACAGGCAGGTTTACGATTTGATCAATGAGTTGTGGAACTCCTATCAATTTGAAGAAATG
821 Y R Q V Y D L I N E L W N S Y Q F E E M

2530 2540 2550 2560 2570 2580
2521 AGAGAGAAATCACAAGGATCACGCGTTGAGAAACTAGCGCAAGAACTTAAGATTAAATCC
841 R E K S Q G S R V E K L A Q E L K I K S

2590 2600 2610 2620 2630 2640
2581 GGACAGAAGAATAATTTGGAAGGACATGTCCTGTTTAACTCGGTATACGGTTCCATGGTC
861 G Q K N N L E G H V L F N S V Y G S M V

2650 2660 2670 2680 2690 2700
2641 TATCCTTACGACAAGCATAGAATTAGAGAGGCCGTTGCTGCGCTGAAAAAACTTCTGACG
881 Y P Y D K H R I R E A V A A L K K L L T

2710 2720 2730 2740 2750 2760
2701 AGTGACTCCAAACTGAAAACAACTGCATTTAACAATTTCGAAAAGATAGTGAGCTTCCCG
901 S D S K L K T T A F N N F E K I V S F P

2770 2780 2790 2800 2810 2820
2761 ATGGAAATGGGCGTGCCGTTCGTCTATTCTTTCGAGCTGCCAGTATTTGTAAAAAGTGAA
921 M E M G V P F V Y S F E L P V F V K S E

2830 2840 2850 2860 2870 2880
2821 ATTAACTTTAAAAAAGGAGAACCCATCACCTCGAGGAGTGGCGTTTACGAAACGTTATTC
941 I N F K K G E P I T S R S G V Y E T L F

2890 2900 2910 2920 2930 2940
2881 TGCAATAGAGTACAGAAGCGGTTTGGTTTCATAGCACCTTTCGAGTATCAGAATTATATT
961 C N R V Q K R F G F I A P F E Y Q N Y I

2950 2960 2970 2980 2990 3000
2941 GCTGGTATTGACAAAAATGGAATAATGCGAGTACCTCTAAAATACGAAACCAATATTGAT
981 A G I D K N G I M R V P L K Y E T N I D

3010 3020 3030 3040 3050 3060
3001 ATAAAACAGAAAAACTTTGCATTGAAGATTCATCCGAACATACCGCAATCTGGAACGAGT
1001 I K Q K N F A L K I H P N I P Q S G T S

3070 3080 3090 3100 3110 3120
3061 ACTGGATTAACACATTATAGTGTCGTTCCCTTCACCACGCGACAAAATATCTTTAATCTT
1021 T G L T H Y S V V P F T T R Q N I F N L

3130 3140 3150 3160 3170 3180
3121 CAACCAGTATCTAATGAGGGTAATACGCGTCCTGTGATAACCTCAGAAATACATAAAATG

```
1041     Q  P  V  S  N  E  G  N  T  R  P  V  I  T  S  E  I  H  K  M

             3190       3200       3210       3220       3230       3240
3181     ACAAAAGAAAAAGGTCCATTTTCTATCAAAATAGAATCTGATACCACGAAAAAAGAATCA
1061     T  K  E  K  G  P  F  S  I  K  I  E  S  D  T  T  K  K  E  S

             3250       3260       3270       3280       3290       3300
3241     GTCTTGGAAGATATCGTTACGGGAATCTCGAAATCATCTAATTCTAATAACGAGCGTTAT
1081     V  L  E  D  I  V  T  G  I  S  K  S  S  N  S  N  N  E  R  Y

             3310       3320       3330       3340       3350       3360
3301     ATGAAAATAGACACAACATTTGAGTCTAAGCAAGTAGCAAAGTGTGAGATACAAATCGAC
1101     M  K  I  D  T  T  F  E  S  K  Q  V  A  K  C  E  I  Q  I  D

             3370       3380       3390       3400       3410       3420
3361     ATGACGTTCGATGCAGTGACAATTCACGGTAAGAATCAGCAACCATCGCATAAAGAGATG
1121     M  T  F  D  A  V  T  I  H  G  K  N  Q  Q  P  S  H  K  E  M

             3430       3440       3450       3460       3470       3480
3421     CAACACCACAGTAAACTGGATTGGAAACCAAACAGTAAGGAAAGAAGAGAAGAAATTGTA
1141     Q  H  H  S  K  L  D  W  K  P  N  S  K  E  R  R  E  E  I  V

             3490       3500       3510       3520       3530       3540
3481     AATGTCCTCAGCGCAGGTCTCAAGTCGGGTACAGTCTTTGTAGCGGACGTAAGTTTCAGT
1161     N  V  L  S  A  G  L  K  S  G  T  V  F  V  A  D  V  S  F  S

             3550       3560       3570       3580       3590       3600
3541     CTCCCGAGGTTACAAGACAACACTTATGTTTTTACTGTCGGCAGCGTGAGAAGTAATATA
1181     L  P  R  L  Q  D  N  T  Y  V  F  T  V  G  S  V  R  S  N  I

             3610       3620       3630       3640       3650       3660
3601     GACCAGAAATTAAGACATTACTTTTATGTTAATACTAATGCAGCGCAAGAAGTAAAATAT
1201     D  Q  K  L  R  H  Y  F  Y  V  N  T  N  A  A  Q  E  V  K  Y

             3670       3680       3690       3700       3710       3720
3661     GAACTTTGTTACTCACAAGAAGTACAATATGCATATCCTACTCCTCTCAATTTCGAATAC
1221     E  L  C  Y  S  Q  E  V  Q  Y  A  Y  P  T  P  L  N  F  E  Y

             3730       3740       3750       3760       3770       3780
3721     GCCATTAATAACGAACCAAAAGATAAATTAAAGGGTGTATTGCGATATGGAAGAACCTGC
1241     A  I  N  N  E  P  K  D  K  L  K  G  V  L  R  Y  G  R  T  C

             3790       3800       3810       3820       3830       3840
3781     AATACAGGAAATGAAATTGTTATCACCGGAAGCTCCTCACAAAGTCCACAACTGAGAGAT
1261     N  T  G  N  E  I  V  I  T  G  S  S  S  Q  S  P  Q  L  R  D

             3850       3860       3870       3880       3890       3900
3841     ATGATAGAGAACTCCAGCATTACCAAACAATGTATGGAAGAGATTCAAAAGGGAAAGAAA
1281     M  I  E  N  S  S  I  T  K  Q  C  M  E  E  I  Q  K  G  K  K

             3910       3920       3930       3940       3950       3960
3901     TCTGTGCGAACTTGCAATAAGGCTACTGACGTTGCCCAAGTGAGGGATCAGCTAAACTTT
1301     S  V  R  T  C  N  K  A  T  D  V  A  Q  V  R  D  Q  L  N  F

             3970       3980       3990       4000       4010       4020
3961     CATATTGATGCATCCCAGCTTTCTGAAATACGCCAGAAATATGACCAGGTGATCGGTCTC
1321     H  I  D  A  S  Q  L  S  E  I  R  Q  K  Y  D  Q  V  I  G  L

             4030       4040       4050       4060       4070       4080
4021     CTCAATTATACGAATTTATCACAATACAATGTTCAGCAAAACTCTGAAACCAACACCATC
1341     L  N  Y  T  N  L  S  Q  Y  N  V  Q  Q  N  S  E  T  N  T  I

             4090       4100       4110       4120       4130       4140
4081     GTTGTACAAAACCCATGGGTGATGGTACCAACCGTACAAGAGCCGTGGTACCGTTGGGCC
1361     V  V  Q  N  P  W  V  M  V  P  T  V  Q  E  P  W  Y  R  W  A

             4150       4160       4170       4180       4190       4200
4141     ATTAAACCTTCAGAATCACAAAGGCAAAGTGAAATTGATGTCCTGTTAGACGAAGTATCA
1381     I  K  P  S  E  S  Q  R  Q  S  E  I  D  V  L  L  D  E  V  S

             4210       4220       4230       4240       4250       4260
```

4201 CAACCTTCGTGCACTCTCGATAACGACAAGATTCTTACTTTTGATAACCAGCTCTATAAC
1401 Q P S C T L D N D K I L T F D N Q L Y N

4270 4280 4290 4300 4310 4320
4261 GTGCAACTAGGAAAATGTAAGCACGTGCTTTTAACCACCTATCCACAAGATTCCCATAAT
1421 V Q L G K C K H V L L T T Y P Q D S H N

4330 4340 4350 4360 4370 4380
4321 CGTAGAAATTATATTCCGGAAAGCTCGAAAGTGGCTGTCTTAGCTAAAGATACGGACAAT
1441 R R N Y I P E S S K V A V L A K D T D N

4390 4400 4410 4420 4430 4440
4381 GACAGCAGAAATGTTTATGTATGGCTGGGCAATCTAGAAATTGAACTGAAGAAAGTGGGT
1461 D S R N V Y V W L G N L E I E L K K V G

4450 4460 4470 4480 4490 4500
4441 AATGATCTAAAAGTTGCAATAAACGGACAGAACGTCGAGATACCGGAGAAAGGCCATCAA
1481 N D L K V A I N G Q N V E I P E K G H Q

4510 4520 4530 4540 4550 4560
4501 GAAAGCAATGGAAATGAAATCATTTTCGAAATCGTGCAATTACCAGACGGATCCCTCTCT
1501 E S N G N E I I F E I V Q L P D G S L S

4570 4580 4590 4600 4610 4620
4561 GTCATTTCAGAGAAGTATGGAATAACCGTCATATTCGACGGAAAACACGTTCGATTATAT
1521 V I S E K Y G I T V I F D G K H V R L Y

4630 4640 4650 4660 4670 4680
4621 GCAAATGGTGCAACATATCGTAATGCCATACGTGGTCTCTGCGGCAACTACGACTCCAGG
1541 A N G A T Y R N A I R G L C G N Y D S R

4690 4700 4710 4720 4730 4740
4681 CGTGATAACGATTTCCTCACTCCTAAGAACTGTCTCTTGACGAAACCGGAAGAATTTGCC
1561 R D N D F L T P K N C L L T K P E E F A

4750 4760 4770 4780 4790 4800
4741 GCTACCTACGCTATGACAAACGAGAATTGCCAAGGACCTGCTCCGGAAAATAAACGAAAA
1581 A T Y A M T N E N C Q G P A P E N K R K

4810 4820 4830 4840 4850 4860
4801 GCCGAAGGAGCCATGTGCATTGAAGTGCCAGAGCAGCAACAGATGAACGTTATCAGCGAC
1601 A E G A M C I E V P E Q Q Q M N V I S D

4870 4880 4890 4900 4910 4920
4861 AGAGAAGCGGGTAGAATGATGACAGAGGGCGGAAATTGGGGCTACCATCAGTCTAATCGC
1621 R E A G R M M T E G G N W G Y H Q S N R

4930 4940 4950 4960 4970 4980
4921 AAGAAAGAACATGGACAAGATAGTAAAAGAGGTCACGGTCATAAAAAATACAATCAGAAA
1641 K K E H G Q D S K R G H G H K K Y N Q K

4990 5000 5010 5020 5030 5040
4981 GACTCGCAGGAGGGTGGATCAAACGAGAGTCAATATAGGAAGAAACACAACATTGTCTAT
1661 D S Q E G G S N E S Q Y R K K H N I V Y

5050 5060 5070 5080 5090 5100
5041 CGCACGAGAGTTGTAGAAATGGACGATAAAATCTGTTTCACTACTACACCTGTACCAGGA
1681 R T R V V E M D D K I C F T T T P V P G

5110 5120 5130 5140 5150 5160
5101 TGCCTCCAAGATACCAGGCCCGTAGAAAGAGTTCCGAAGAAATATGATCTTTATTGCCTG
1701 C L Q D T R P V E R V P K K Y D L Y C L

5170 5180 5190 5200 5210 5220
5161 TCGAAGAACAATGAGTCTATGGACCTCAAACGTAGGGTCGAGGAAGGCGCTAAGCCAGAT
1721 S K N N E S M D L K R R V E E G A K P D

5230 5240 5250 5260 5270 5280
5221 TTTACTCAAAAGCCTGTCAACAAGATACAAAACTTCCAAATTCCTGTCTCTTGCAGCGCC
1741 F T Q K P V N K I Q N F Q I P V S C S A

5281 GCATAA
1761 A *
