# Supplementary material for: Comparative Analyses of Reproductive Caste Types Reveal Vitellogenin Genes Involved in Queen Fertility in Solenopsis invicta
Source: Int J Mol Sci. 2023 Dec 5;24(24):17130. doi: 10.3390/ijms242417130 (PMC10743176; doi:10.3390/ijms242417130)
Supplement: Supplementary file 1 [file ijms-24-17130-s001.zip › Table S1/Summary of the sequencing data assembly.pdf]

Summary of the sequencing data assembly

Transcriptome description	Sample name								
	QA-1	QA-2	QA-3	FA-1	FA-2	FA-3	MA-1	MA-2	MA-3
Total raw reads	45828444	40501038	46660864	42488336	45187862	44771152	43383638	48468592	47269852
Total clean reads	44571990	39221358	44993278	41051176	43558014	43233780	41475132	47431276	45444940
Total bases(G)	6.87	6.08	7	6.37	6.78	6.72	6.51	7.27	7.09
GC content(%)	42.22	42.73	42.24	42.5	42.48	42.54	42.36	41.54	42.66
Clean Read Q20(%)	96.53	96.51	96.68	96.58	96.97	96.72	96.62	96.74	96.6
Clean Read Q30(%)	90.84	90.82	91.15	90.97	91.81	91.27	91.14	91.32	91.06
Total mapped reads	41471975	35213158	41921839	38258706	40848687	40501305	38327320	43308707	42555634
Total mapped rated(%)	93.04	89.78	93.17	93.2	93.78	93.68	92.41	91.31	93.64
Total Uniquely mapped reads	40703838	34584062	41167963	37684412	40170948	39835014	37581958	42528596	41738146
Total Uniquely mapped rate(%)	91.32	88.18	91.5	91.8	92.22	92.14	90.61	89.66	91.84
Total Multiple mapped reads	768137	629096	753876	574294	677739	666291	745362	780111	817488
Total Multiple mapped rate(%)	1.72	1.6	1.68	1.4	1.56	1.54	1.8	1.64	1.8
