# Supplementary material for: Comparative Analyses of Reproductive Caste Types Reveal Vitellogenin Genes Involved in Queen Fertility in Solenopsis invicta
Source: Int J Mol Sci. 2023 Dec 5;24(24):17130. doi: 10.3390/ijms242417130 (PMC10743176; doi:10.3390/ijms242417130)
Supplement: Supplementary file 1 [file ijms-24-17130-s001.zip › Table S5/Primers used in this study.pdf]

Primers used in this study

Gene	Upstream Primer (5’ -3’)	Downstream Primer (5’ -3’)	Purpose
SiVg2-1	CTGCAAGTCGATACTCAGGGCG	GGATGTTGGGAGCCAGGAAAAT	Gene cloning
SiVg2-2	GTTCTAGCAGCTCCAGCAGCTC	ACGAACTTCGTGAGCCTCGTTT	
SiVg2-3	GGCGTCAACGTACCAACGTACA	TGGCGATTAACGCTTGGAAAGTG	
SiVg2-4	GCGAATGAAGACGGTCTGCCG	AGACTTCGCCTGACTTGATACCT	
SiVg2-5	ATACGGTGACAATTGGCGGTAA	ACTGTGATTCCGAACTTGGTCC	
SiVg2-6	CTGCTGTTTGCATCACCAGTCA	TCATGCGGCGCTGCAAGA	
SiVg3-1	ATGTGGTTCCCTGTCGTCCTT	AGACAGTACCAATGGGAGAATCG	
SiVg3-2	ACGACTCACATAGTTATCACGGG	TACCGGATAGTAAGATGGGACGA	
SiVg3-3	TACGTAAATGTGTCCGCACCTCT	ACCATGGAACCGTATACCGAGTT	
SiVg3-4	AAATCACAAGGATCACGCGTTGA	TCCGCTACAAAGACTGTACCCGA	
SiVg3-5	TCAGCAACCATCGCATAAAGAGA	GATTGCCCAGCCATACATAAACA	
SiVg3-6	TCGTGCACTCTCGATAACGACAA	TTATGCGGCGCTGCAAGA	
SiVg2	CCTCCGTGGAAGCAGGATAC	TTTCGTCGCTCATTTCGTCG	RT-qPCR
SiVg3	CCGGAAGCTCCTCACAAAGT	TGATCCCTCACTTGGGCAAC	
ef1-beta	CCTGAAGACCGATAAGGGCAT	GATTGTTGTGTTGGTGGTTTCC	
ds*SiVg2*	TAATACGACTCACTATAGGGCGT CAGTGGCAAATGCGAGG	TAATACGACTCACTATAGGGAGGT TTCCGGTTGATTCGAGGT	dsRNA synthesis
ds*SiVg3*	TAATACGACTCACTATAGGGAATC ACAAGGATCACGCGTTGAG	TAATACGACTCACTATAGGGTGCG GTATGTTCGGATGAATCTT	
dsEGFP	TAATACGACTCACTATAGGGGAC GTAAACGGCCACAAGTTC	TAATACGACTCACTATAGGGGTAC AGCTCGTCCATGCCG	
