# Supplementary figures and images for: Comparative Analyses of Reproductive Caste Types Reveal Vitellogenin Genes Involved in Queen Fertility in Solenopsis invicta
Source: Int J Mol Sci. 2023 Dec 5;24(24):17130. doi: 10.3390/ijms242417130 (PMC10743176; doi:10.3390/ijms242417130)

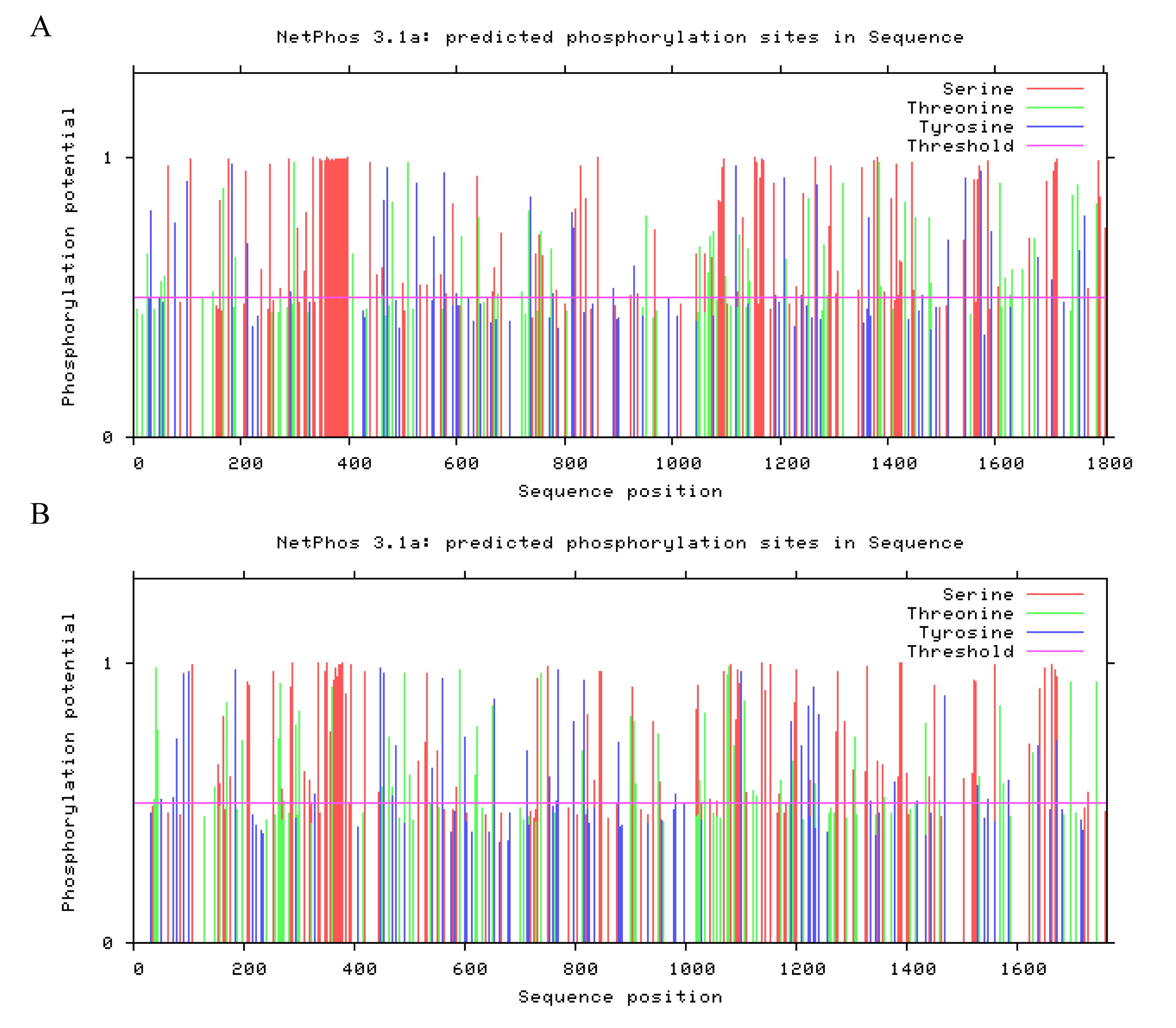

Supplement: Supplementary file 1 [file ijms-24-17130-s001.zip › Fig. S2/┴╫╦ß╗»╬╗╡π╘ñ▓Γ_.tif]

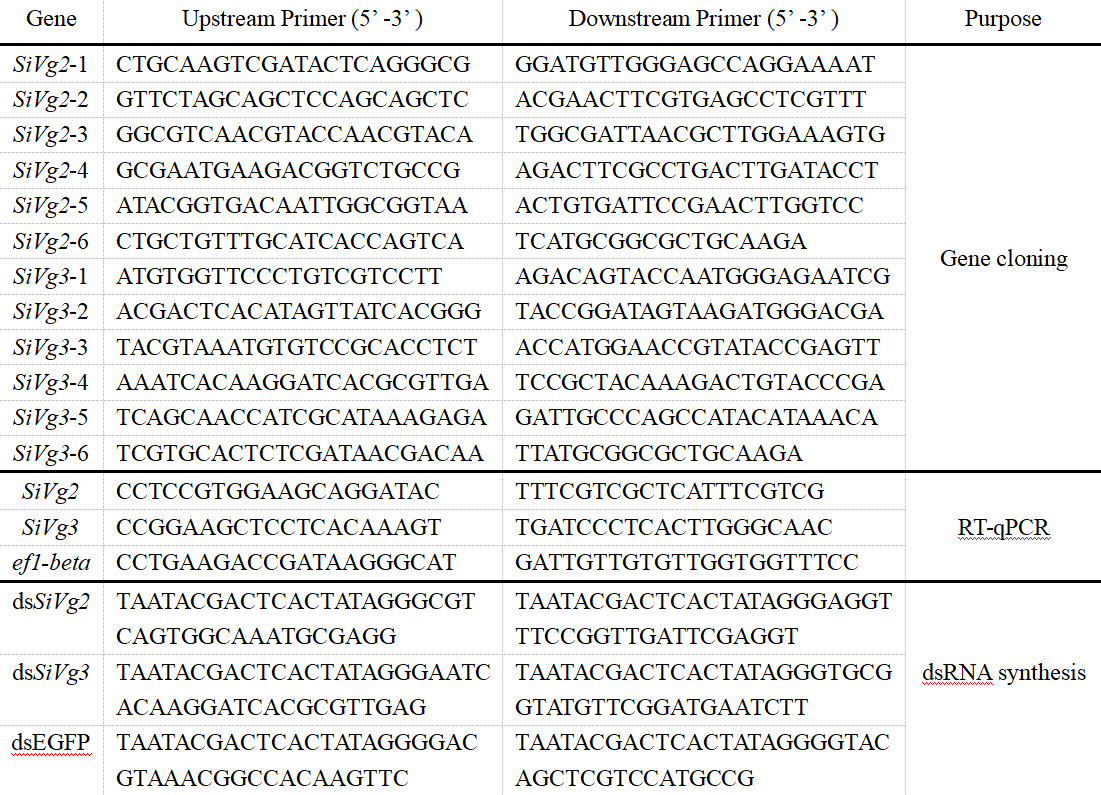

Supplement: Supplementary file 1 [file ijms-24-17130-s001.zip › Table S5/table 1.png]
